# Molecular and thermodynamic determinants of self-assembly and hetero-oligomerization in the enterobacterial thermo-osmo-regulatory protein H-NS

**DOI:** 10.1093/nar/gkae090

**Published:** 2024-02-10

**Authors:** Bincy Lukose, Takahiro Maruno, Mohammed A Faidh, Susumu Uchiyama, Athi N Naganathan

**Affiliations:** Department of Biotechnology, Bhupat & Jyoti Mehta School of Biosciences, Indian Institute of Technology Madras, Chennai 600036, India; Department of Biotechnology, Osaka University, Japan; Department of Biotechnology, Bhupat & Jyoti Mehta School of Biosciences, Indian Institute of Technology Madras, Chennai 600036, India; Department of Biotechnology, Osaka University, Japan; Department of Biotechnology, Bhupat & Jyoti Mehta School of Biosciences, Indian Institute of Technology Madras, Chennai 600036, India

## Abstract

Environmentally regulated gene expression is critical for bacterial survival under stress conditions, including extremes in temperature, osmolarity and nutrient availability. Here, we dissect the thermo- and osmo-responsory behavior of the transcriptional repressor H-NS, an archetypal nucleoid-condensing sensory protein, ubiquitous in enterobacteria that infect the mammalian gut. Through experiments and thermodynamic modeling, we show that H-NS exhibits osmolarity, temperature and concentration dependent self-association, with a highly polydisperse native ensemble dominated by monomers, dimers, tetramers and octamers. The relative population of these oligomeric states is determined by an interplay between dimerization and higher-order oligomerization, which in turn drives a competition between weak homo- versus hetero-oligomerization of protein-protein and protein-DNA complexes. A phosphomimetic mutation, Y61E, fully eliminates higher-order self-assembly and preserves only dimerization while weakening DNA binding, highlighting that oligomerization is a prerequisite for strong DNA binding. We further demonstrate the presence of long-distance thermodynamic connectivity between dimerization and oligomerization sites on H-NS which influences the binding of the co-repressor Cnu, and switches the DNA binding mode of the hetero-oligomeric H-NS:Cnu complex. Our work thus uncovers important organizational principles in H-NS including a multi-layered thermodynamic control, and provides a molecular framework broadly applicable to other thermo-osmo sensory proteins that employ similar mechanisms to regulate gene expression.

## Graphical Abstract

**Graphical Abstract:**
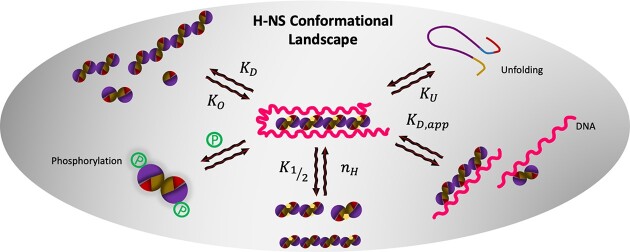

## Introduction

The resilience and adaptability of bacterial systems are a consequence of environmental sensing mechanisms at the molecular level. H-NS (histone-like or heat-stable nucleoid structuring protein), a multi-faceted stress response system and a known ‘sensor’ of environment, dynamically modulates self-oligomerization and hetero-oligomerization involving co-repressors, other nucleoid-associated proteins (NAPs) and DNA, thus regulating genomic compaction, xenogenic silencing, transcription and even pathogenicity via mechanisms that are still being understood (1–11). Remarkably, H-NS controls nearly ∼5% of ORFs in the *Escherichia coli* genome (12). It is abundantly expressed in uropathogenic *E. coli*, *Salmonella typhimurium* and *Shigella* sp., with homologs in *E. coli* (termed StpA), *Pseudomonas aeruginosa* (MvaT) and *Mycobacterium tuberculosis* (Lsr2) (13–20). H-NS also contributes to multi-drug resistance (MDR) by regulating the expression of multidrug efflux transporters, (21,22) while H-NS degradation enables the expression of genes essential for mammalian gut colonization (23).

**Figure 1.**
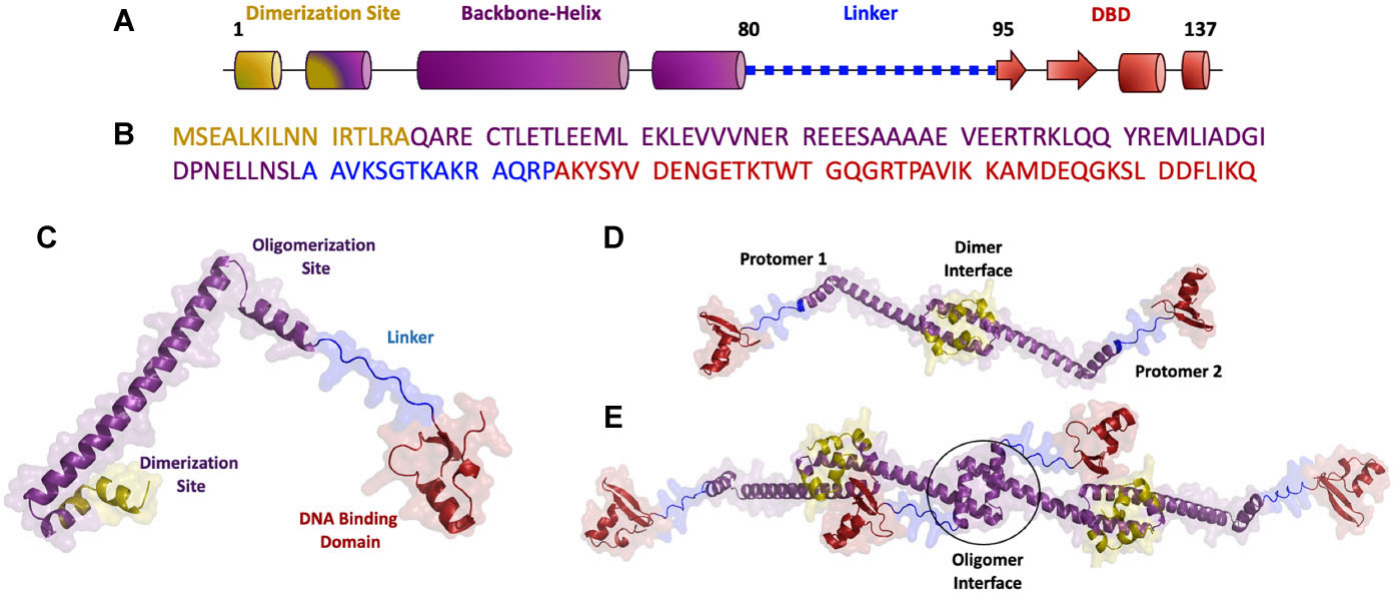
H-NS structural organization and assembly modes. (**A, B**) Cartoon of H-NS showing the helix and strands as cylinders and arrows, respectively. The sequence shown in panel B follows the color code of panel A. (**C**) Structural model of H-NS as predicted by AlphaFold-ColabFold server with a C_α_-RMSD of 0.8 Å and 2.1 Å with the experimental structures of the dimerization (PDB id: 3NR7) (19) and DNA-binding domains (1HNS) (31), respectively. (**D**, **E**) Predicted structural models of H-NS dimer (panel D) and tetramer (panel E).

Structurally, H-NS and H-NS family of proteins are primarily helical in nature with ∼130–140 amino acids. The structure of H-NS protomer can be sub-divided into four distinct regions (Figure 1A–C, Supporting Supplementary Figure S1): the N-terminal dimerization site (the ‘head’), the long backbone helix, the flexible linker region and the DNA-binding domain (DBD; Figure 1B, C) (19,24–26). The helical N-terminal dimerization site enables dimerization through head-to-head contacts (Figure 1D). The oligomerization site located at the C-terminal end of long backbone helix enables higher-order oligomerization via tail-to-tail contacts (Figure 1E), which is a critical step required for modulating gene expression (27). H-NS thus self-associates to form higher-order oligomers ranging anywhere between dimer to 16-mer but whose stoichiometry appears to depend on the source organism (i.e. paralogs and orthologs), the construct employed (small domains, their variants and the full length proteins), experimental conditions and protein concentrations used (8,15,19,24,25,28,29).

The molecular features of H-NS that determine functional outcomes are varied and span a range of processes. For example, it has been proposed that at elevated temperatures (say, 310 K, which is the body temperature of a mammalian host), gene repression is eliminated by the disassembly of H-NS oligomers, (4) while the oligomerization is enhanced by macromolecular crowding (30) which presumably acts as a protective mechanism under high extracellular osmolarity. H-NS also forms a heteromer with StpA (a paralog) stabilizing it, which is otherwise degraded by the Lon protease (15,32). H-NS binds DNA via the DBD in a non-sequence specific manner, but with a preference for AT-rich regions, (26) forming both linear (a lattice of H-NS on DNA) and bridged H-NS assemblies (sandwiched between two DNA duplexes) (33,34). Similarly, the H-NS orthologs Lsr2 (from *M. tuberculosis*) and MvaT (*P. aeruginosa*) have been shown to form filamentous but bridged structures (35,36). Apart from the assembly-disassembly equilibrium, the composition and patterning of charges on the linker region have been proposed to govern the polymerization of H-NS along DNA, eventually determining the extent of bacterial mobility (37,38). In addition to homo- and hetero-oligomer formation, conformational diversity is also an intrinsic feature of H-NS family proteins, with MvaT sampling at least two conformational states in the dimeric assembly (18).

Here, we investigate the thermodynamics of the multi-layered regulation mechanism involving H-NS from uropathogenic *E. coli*, with specific insights into the stoichiometry of self-assembly and show that the H-NS native ensemble exists in an equilibrium between monomers, dimers, tetramers and octamers. In addition to folding-unfolding equilibrium, there is a strong temperature dependence on the size and polydispersity of the H-NS oligomeric assembly under physiological salt concentrations, while higher salt modulates the cooperativity of unfolding and disassembly. We find that higher-order oligomerization is critical for DNA binding, as an engineered phosphomimetic mutant that populates solely the dimeric state is unable to bind DNA strongly. Binding modes to DNA are switched in the presence of the co-repressor Cnu, highlighting hitherto unreported allosteric connectivity between oligomerization and dimerization sites on H-NS.

## Materials and methods

### Over-expression and purification of *E. coli* H-NS

BL21(DE3) cells containing pTXB1 vector (IMPACT™ from New England Biolabs) with H-NS gene corresponding to the protein sequence MSEALKILNNIRTLRAQARECTLETLEEMLEKLEVVVNERREEESAAAAEVEERTRKLQQYREMLIADGIDPNELLNSLAAVKSGTKAKRAQRPAKYSYVDENGETKTWTGQGRTPAVIKKAMDEQGKSLDDFLIKQ were grown at 37°C and 180 rpm. The cells were induced with 1 mM IPTG at an OD_600nm_ ∼0.8 and incubated for 4 hours at 37°C to facilitate the overexpression of H-NS:intein:CBD (chitin-binding domain) fusion protein. Following incubation, the cells were harvested by centrifugation at 9500 rpm for 3 minutes at a temperature of 4°C. The pellet corresponding to one litre culture cells was subsequently resuspended in 80 ml lysis buffer consisting of 20 mM sodium phosphate buffer, pH 8, 500 mM NaCl and 1 mM EDTA and lysed. Following sonication, the cell lysate was centrifuged at 11000 rpm for 90 minutes at 4°C. The clear supernatant was loaded to a fresh chitin column pre-equilibrated with the lysis buffer at a flow rate of 0.5–1 ml/min. In order to remove non-specifically bound proteins and impurities from the chitin, the resin underwent a thorough washing process using two sets of 10-column volumes of buffer. The first wash (wash 1) comprised 20 mM sodium phosphate buffer at pH 8, 1 M NaCl and 1 mM EDTA, while the second wash (wash 2) was with the buffer consisting of 20 mM sodium phosphate buffer at pH 8, 300 mM NaCl and 1 mM EDTA. Subsequently, the resin was washed using the cleavage buffer, containing 20 mM sodium phosphate buffer at pH 8, 300 mM NaCl, 1 mM EDTA and 100 mM β-mercaptoethanol, to initiate the intein-mediated cleavage reaction. The cleaved product was eluted with elution buffer (cleavage buffer devoid of β-mercaptoethanol) in 5 ml fractions. Approximately 80% cleavage efficiency was obtained after 40 hours of incubation at room temperature. The eluted fractions were again passed through a fresh chitin column pre-equilibrated with the elution buffer to remove the intein:CBD impurities and flowthrough was collected. The H-NS protein fractions were further purified through a HiLoad 26/600 Superdex 200 pg preparative size exclusion chromatography column. Prior to this purification step, the column was equilibrated with 150 mM ammonium acetate buffer at pH 8.0. The purity of the eluted protein fractions was assessed using a 16.5% SDS-PAGE gel. The fractions containing the purified H-NS protein were combined, subjected to lyophilization, and stored at a temperature of -80°C for further use. The mutants Y61E, H-NSm (W109F) and Y61Em (Y61E/W109F) were purified following identical protocols. All experiments were carried out in either 150 mM buffer (pH 7, 20 mM sodium phosphate plus NaCl to result in an effective ionic strength of 150 mM) or 300 mM buffer (pH 7, 20 mM sodium phosphate plus NaCl to result in an effective ionic strength of 300 mM).

### Size-exclusion chromatography

Different concentrations (15, 50, 100 and 200 μM) of H-NS and Y61E mutant were dissolved in 300 mM buffer (pH 7, 20 mM sodium phosphate buffer plus NaCl). The protein solutions (1 ml) were then injected into a pre-equilibrated HiLoad 26/600 Superdex 200 pg column at a flow rate of 2 ml/min. The elution profiles obtained from the column were then analyzed. A higher ionic strength buffer was employed to completely eliminate non-specific interactions of these highly-charged proteins with the column matrix.

### Sedimentation velocity-analytical ultracentrifugation experiments

SV-AUC experiments for H-NS and the Y61E mutant were conducted at 20°C using Optima AUC (Beckman Coulter) with a UV-Vis detection system at both 150 and 300 mM ionic strength buffer solutions. A volume of 390 μl of the sample solution was loaded into the sample sector equipped with sapphire windows and a 12-mm double-sector charcoal-filled Epon centerpiece, and 400 μl of a corresponding solvent was loaded into each reference sector. The rotor speed was set at 50000 rpm. Data were collected every 120 s with a radial increment of 10 μm. Detection wavelengths were set at 232, 239, 280, 287, 290 and 294 nm for 15, 50, 90, 100, 125 and 200 μM sample solutions, respectively, regardless of the ionic strength. The data were analyzed using the continuous c(s) distribution of program SEDFIT (version 16.2b) (39) fitting for the frictional ratio, meniscus, time-invariant noise, and radial-invariant noise and using a regularization level of 0.68. The sedimentation coefficient ranges of 0–15 *S* were evaluated with a resolution of 150. The partial specific volume of the samples, the buffer density, and buffer viscosity were calculated using program SEDNTERP (40). Figures of *c*(*s*) distribution were generated using program GUSSI (version 1.3.2) (41).

### Steady-state tryptophan anisotropy

The self-assembly of H-NS as a function of protein concentration was investigated by monitoring changes in fluorescence anisotropy specifically attributed to the tryptophan residue (W109) located in its C-terminal DNA binding domain. To study this, a tryptophan null variant of H-NS (W109F; termed H-NSm) was generated. The experiments were carried out in a 150 mM ionic strength buffer at 25 and 37°C. Here, a 2 μM wild-type H-NS solution was excited at 295 nm in the presence of varying concentrations of the H-NS tryptophan variant (H-NSm) ranging from 30 nM to 50 μM (final volume of 2.2 ml). Changes in anisotropy were monitored after a five-minute equilibration period at each temperature. Excitation of the H-NS sample was performed at 295 nm, and the emitted light at 340 nm was collected using a Chirascan-plus qCD instrument with a fluorescence polarization (FP) accessory equipped with a Peltier unit (Applied Photophysics, UK), and the data was acquired at every 20 s time per point. A cut-off filter of 320 nm was placed before the FP detector to filter the emitted photons.

### Far-UV circular dichroism

Equilibrium thermal unfolding experiments were monitored by far-UV CD in 150 mM buffer, pH 7 in a Jasco J-815 spectropolarimeter equipped with a Peltier system at a protein concentration of 5 μM in one mm pathlength quartz cuvettes (total volume of 350 μl). Spectra were recorded from 5 to 95°C in 5°C increments, between 200 and 250 nm. The data were acquired at 1 nm intervals at a bandwidth of 2 nm, and with a scan speed of 10 nm/min.

### Differential scanning calorimetry

DSC experiments were carried out in 150 and 300 mM ionic strength conditions at a range of protein concentrations spanning 15–200 μM (sample cell volume of 142 μl). The samples were extensively degassed at 25°C before loading into the calorimetric cell. Calorimetric measurements were performed using a MicroCal VP-Capillary (Malvern, UK) automated differential scanning calorimeter, scanning temperatures from 5 to 95°C at a scan-rate of 1 K/min. Prior to each protein scan, several buffer scans were taken to monitor thermal drift. The calorimetric cells were maintained at a high pressure (∼60 psi) during operation to prevent water loss from the samples at elevated temperatures.

### DNA binding assays

The interaction between H-NS variants and DNA was assessed by monitoring the steady-state anisotropy of a 100 bp double-stranded DNA (29) with an AT-rich sequence labeled with Cy3 at the 5′ end (IDT Primers). The DNA concentration was set at 40 nM and titrated with various concentrations of the protein (H-NS or the Y61E mutant) ranging from approximately 1 nM to 50 μM, in both 150 and 300 mM buffer in a 1 cm × 1 cm pathlength cuvette (final volume of 3.75 ml). Changes in anisotropy were measured after a 5-min equilibration period at each titration step. The Cy3-dye was excited at 555 nm, and the emitted light at 600 nm was collected using a Chirascan-plus qCD instrument equipped with a fluorescence polarization accessory. Data were acquired at intervals of 20 s time per point, with excitation and emission bandwidths set at 12 nm. The PMT detector voltage was adjusted for each experimental condition. Anisotropy measurements were acquired in the presence of the co-repressor Cnu, with fixed concentrations of 5 and 50 μM.

### Fluorescence titration assays

The extent and nature of H-NS:Cnu interaction was investigated by recording the fluorescence emission spectra of 2 μM Cnu upon excitation at 295 nm at 25 and 37°C in the presence of H-NS mutants (H-NSm or Y61Em which do not have a tryptophan) at concentrations ranging from 30 nM to 50 μM. A 320 nm cut-off filter was placed in front of the detector, and the spectra were collected after equilibrating the samples for 5 min. Data were acquired at an excitation bandwidth of 5 nm and an emission bandwidth of 10 nm. Since both the emission intensity and maximum emission wavelength change on binding, the intensity-averaged wavenumber (‘Signal’)—defined as ${\bar{\nu }}_{av} = {10}^4\frac{{{{\mathrm{\Sigma }}}_\lambda I( \lambda ){\lambda }^{ - 1}}}{{{{\mathrm{\Sigma }}}_\lambda I( \lambda )}}$, where $I( \lambda )$ represents the intensity of fluorescence emission at wavelength $\lambda$—was employed to monitor binding of H-NSm to Cnu.

### Thermodynamic modeling of oligomerization and unfolding

Consider a system defined by the following collection of equilibria involving octamer (${N}_8$), tetramer (${N}_4$), dimer (${N}_2$), folded monomer ($N$) and unfolded monomer ($U$),


(1) \begin{equation*}{N}_8 \mathbin{\lower.3ex\hbox{$\buildrel\textstyle\leftharpoonup\over {\smash{\rightharpoondown}}$}} 2{N}_4\ , {N}_4 \mathbin{\lower.3ex\hbox{$\buildrel\textstyle\leftharpoonup\over {\smash{\rightharpoondown}}$}} 2{N}_2\ , {N}_2 \mathbin{\lower.3ex\hbox{$\buildrel\textstyle\leftharpoonup\over {\smash{\rightharpoondown}}$}} 2N\ , N \mathbin{\lower.3ex\hbox{$\buildrel\textstyle\leftharpoonup\over {\smash{\rightharpoondown}}$}} U\end{equation*}

The corresponding equilibrium constants for oligomerization (*O*), dimerization (*D*) and unfolding (*U*) are


(2) \begin{eqnarray*}\frac{1}{{{K}_O}} = {K}_o = \frac{{\left[ {{N}_4} \right]}}{{{{\left[ {{N}_2} \right]}}^2}} = \frac{{\left[ {{N}_8} \right]}}{{{{\left[ {{N}_4} \right]}}^2}}, \frac{1}{{{K}_D}} = {K}_d = \frac{{\left[ {{N}_2} \right]}}{{{{\left[ N \right]}}^2}},\ {K}_U = \frac{{\left[ U \right]}}{{\left[ N \right]}} \nonumber\\ \end{eqnarray*}

If the equilibrium constants and the corresponding enthalpies of oligomerization (${\mathrm{\Delta }}{H}_O$), dimerization (${\mathrm{\Delta }}{H}_D$) and unfolding (${\mathrm{\Delta }}{H}_U$) are defined at specific temperatures corresponding to ${T}_O$, ${T}_D$ and ${T}_U$ with the latter being the melting temperature, the temperature ($T$) dependence can be extracted from


\begin{equation*}{\mathrm{\Delta }}{S}_X = \frac{{{\mathrm{\Delta }}{H}_X}}{{{T}_X}} + Rln\left( {{K}_X} \right)\end{equation*}

(3) \begin{equation*}{K}_X\left( T \right) = exp\left( {\frac{{ - {\mathrm{\Delta }}{H}_X}}{{RT}} + \frac{{{\mathrm{\Delta }}{S}_X}}{R}} \right)\end{equation*}

where $X = O,\ D,\ U$. The mass-balance equation for a total monomeric protein concentration $C$ is


(4) \begin{equation*}C = 8\left[ {{N}_8} \right] + 4\left[ {{N}_4} \right] + 2\left[ {{N}_2} \right] + \left[ N \right] + \left[ U \right]\end{equation*}

On substituting equation (2) into equation (4) and rearranging, we get the following expression


(5) \begin{eqnarray*} && 8\frac{{K_o^3K_d^4}}{{K_U^8}}{\left[ U \right]}^8 + 4\frac{{{K}_oK_d^2}}{{K_U^4}}{\left[ U \right]}^4 + 2\frac{{{K}_d}}{{K_U^2}}{\left[ U \right]}^2 \nonumber\\ && \quad +\, \left( {1 + \frac{1}{{{K}_U}}} \right)\left[ U \right] - C = 0\end{eqnarray*}

Solving this equation gives [*U*] which can be used to find the total partition function as


\begin{equation*}Z = \frac{{8\left[ {{N}_8} \right] + 4\left[ {{N}_4} \right] + 2\left[ {{N}_2} \right] + \left[ N \right] + \left[ U \right]}}{{\left[ N \right]}}\end{equation*}

\begin{equation*}Z = {Z}_{{N}_8} + {Z}_{{N}_4} + {Z}_{{N}_2} + {Z}_N + {Z}_U\end{equation*}

which in turn can be written in terms of the equilibrium constants,


(6) \begin{eqnarray*}Z\left( T \right) = 8\frac{{K_o^3K_d^4}}{{K_U^7}}{\left[ U \right]}^7 + 4\frac{{{K}_oK_d^2}}{{K_U^3}}{\left[ U \right]}^3 + 2\frac{{{K}_d}}{{{K}_U}}\left[ U \right] + 1 + {K}_U \nonumber\\ \end{eqnarray*}

Note that the equilibrium constants and the unfolded population are temperature dependent and this is not explicitly shown for the sake of clarity. This can then be used to calculate the constant pressure heat capacity for the system at temperature $T$ as


(7) \begin{equation*}{C}_p\left( T \right) = 2RT\left( {\frac{{dln\ Z\left( T \right)}}{{dT}}} \right) + R{T}^2\left( {\frac{{{d}^2ln\ Z\left( T \right)}}{{d{T}^2}}} \right)\end{equation*}

The species-specific partition functions can be used to calculate species fractions, which describe the fraction of the protein in that particular oligomeric state at temperature $T$ as


(8) \begin{equation*}{{\mathrm{\alpha }}}_Y = \frac{{{Z}_Y}}{Z}\end{equation*}

where $Y = {N}_8,{N}_4,\ {N}_2,\ U$. The model effectively employs 8 parameters: three reference temperatures (${T}_O,\ {T}_D,\ {T}_m$), three enthalpies for the three processes (${\mathrm{\Delta }}{H}_O,\ {\mathrm{\Delta }}{H}_D,\ {\mathrm{\Delta }}{H}_U$) and two equilibrium constants (${K}_o$ at ${T}_O$, ${K}_d$ at ${T}_D$). Since ${K}_U$ is defined at the melting temperature for convenience, it equals one. The parameters employed for simulating various scenarios are provided in Supplementary Table S1.

### Block Wako–Saitô–Muñoz–Eaton (bWSME) model

The model construction, ensemble description, and energetics are described in great detail in earlier works (42–44). Briefly, employing specific sequence approximations and an Ising-like binary approach of *0*s and *1*s for unfolded and folded residues or blocks of consecutive residues, respectively, a large ensemble of microstates (or conformations) are generated employing the input structural model. The statistical weight and hence the probability of every microstate is determined by the corresponding free energy which includes contributions from van der Waals interaction energy, Debye–Hückel electrostatics assuming a pH 7 protonation state and 150 mM ionic strength, and implicit solvation, apart from an entropic penalty term for fixing a residue in a native conformation. In the current work, the AlphaFold-ColabFold (45,46) predicted structural model of H-NS is used as an input, which is then used to generate an ensemble of 1,321,642 microstates. The van der Waals interaction energy per native contact was iteratively modulated to reproduce the experimental stability of DBD and hence the full-length protein, while fixing the magnitude of other parameters. The final parameters are: van der Waals interaction energy of –102 J mol^−1^ per native contact identified with a 5 Å heavy-atom cut-off, entropic penalty of –16.5 J mol^−1^ K^−1^ per residue, and a heat capacity change of –0.36 J mol^−1^ K^−1^ per native contact (to model implicit solvation).

## Results

### The oligomeric nature of H-NS

**Figure 2.**
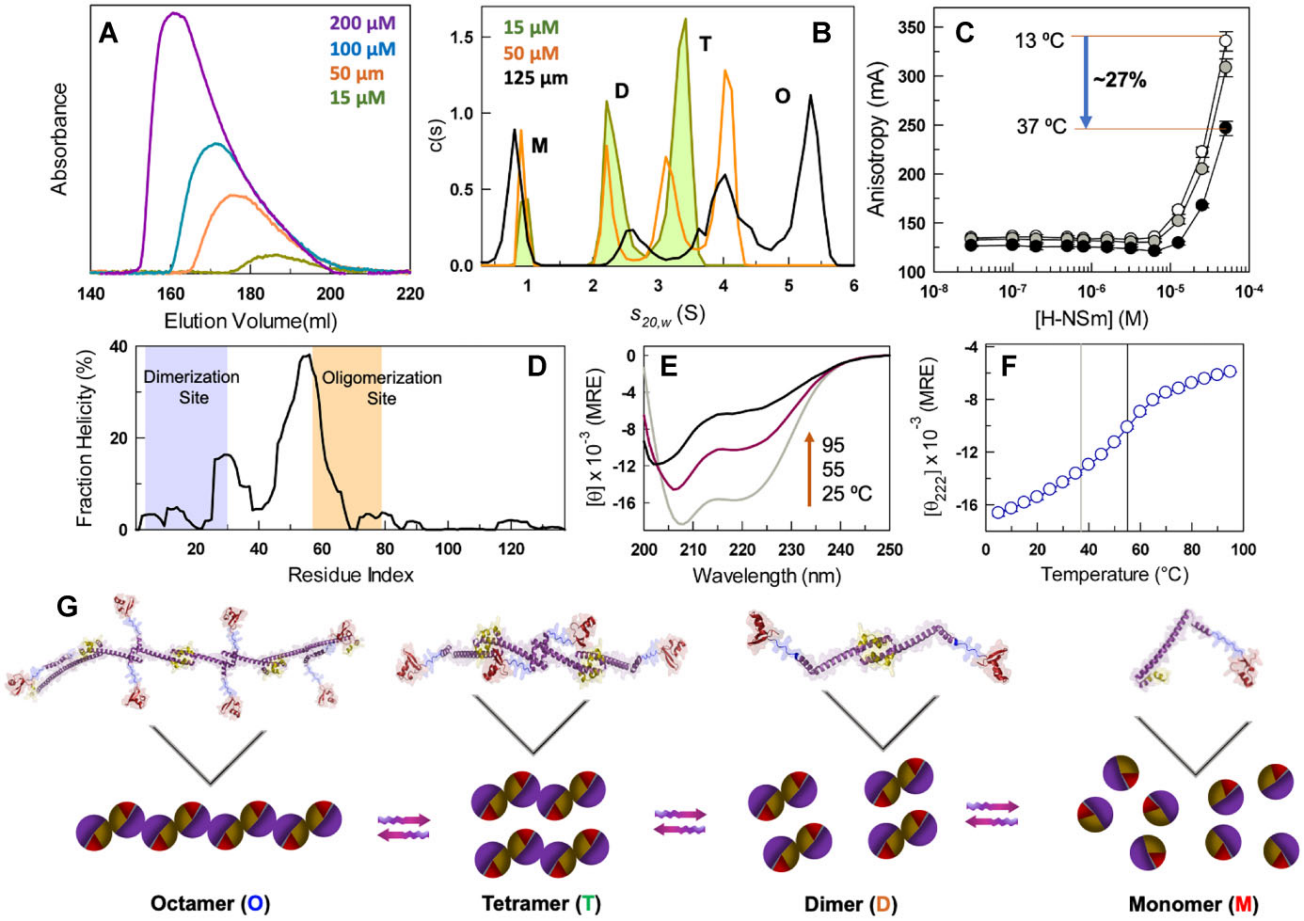
Oligomeric assemblies in the native ensemble of H-NS. All experiments were done at 150 mM ionic strength except for the data in panel A. (**A**) Elution profile of H-NS from a size-exclusion chromatography column at 300 mM ionic strength, pH 7.0 and 25°C. A higher ionic strength was employed to avoid non-specific interactions with the column matrix. (**B**) Distribution of sedimentation coefficients for H-NS at 20°C highlighting distinct peaks corresponding to monomer (M), dimer (D), tetramer (T) and octamer (O) (see Figure 3). Variations in *s*-values are of the order of 0.01 S. (**C**) Tryptophan anisotropy of H-NS when titrated with a variant H-NSm (W109F H-NS) at 13°C (open circles), 25°C (gray filled circles) and 37°C (black circles). The anisotropy rises sharply after 10 μM, but the extent of increase decreases with temperature, hinting at lower oligomerization extents at higher temperatures. All errors reported are standard errors of the mean (SEM) from three replicates. (**D**) Fraction helicity as a function of residue index predicted by AGADIR at 25°C, pH 7.0. (**E**) Far-UV CD spectra at the indicated temperatures reported in mean-residue ellipticity (MRE) units of deg cm^2^ dmol^−1^. (**F**) The mean thermal unfolding curve (from three replicates) monitored by far-UV CD at 222 nm and reported in MRE units. The light and dark vertical lines indicate 37°C and the apparent melting temperature of 55°C. The error bars are smaller than the size of points. (**G**) A schematic representation of the polydisperse H-NS native ensemble. Each circle is a cartoon of a promoter with purple representing the oligomerization site, dark yellow the dimerization site and red being the DBD. This representation is inspired by a similar cartoon depiction of H-NS employed earlier (49).

Size-exclusion chromatography (SEC) experiments reveal broad elution profiles indicative of a highly oligomeric protein (Figure 2A). Specifically, the elution profile at 15 μM exhibits a peak at 185 ml and spans ∼20 ml, while under the same conditions a 200 *μ*M sample displays a peak at 160 ml and a significantly broad profile spanning nearly 50 ml. These are signs that H-NS not only oligomerizes, but does so in a concentration dependent manner forming higher-order oligomeric structures at higher concentrations, mirroring earlier observations on different constructs (15,27). To probe this feature in a quantitative manner, we resorted to analytical ultracentrifugation (AUC) experiments that provide an unbiased view of the oligomeric status at 150 mM ionic strength condition. At 15 *μ*M, the sedimentation coefficient distribution peaks at three different values corresponding to 1, 2.2 and 3.5 S. At 50 μM, an additional higher molecule weight species appears at 4 S, while the population of the lower molecular weight species drop (Figure 2B). This is more obvious at 125 μM concentration where the distributions broaden, shift to the right and with a maximum $s$-value of 5.5. A naïve analysis of the predicted structural models (cartoons in Figures 1 and 2) employing the program HYDROPRO (47) reveals that the monomer, dimer, tetramer and octamer should display $s$-values of 1.1, 1.6, 2.4 and 3.5 S, respectively. On comparison with the observed data, it is evident that the oligomeric nature spans at least till an octamer and that the native ensemble of H-NS is highly polydisperse.

However, four processes make the connection between true oligomeric size and $s$-values non-trivial in H-NS. First, the peak positions of the distributions vary with concentration hinting that the various species are under ‘reaction boundary’ conditions (48). In other words, when the total protein concentration ($C$, corresponding to the protomer) falls in the range corresponding to 0.1${K}_D \leq C \leq 10{K}_D$ with ${K}_D$ being the dissociation constant, the observed $s$-value distribution of the complex would exhibit values which are smaller than the true $s$-value until complete saturation. Second, each of the species observed (from the peak of the $c( s )$ distributions) could be in exchange with a higher-order oligomer, making the assignment of the oligomeric nature challenging based purely on the molecular weight. Third, altered frictional coefficients due to structural changes (say compaction or extension, which will not be captured in predicted structures) can result in experimental $s$-values different from structure-based calculations. Finally, changes in stiffness due to allosteric effects upon forming higher-order oligomers of different molecularities could also result in altered $s$-value distributions.

H-NS harbors only a single tryptophan which is located in the C-terminal DNA-binding domain (DBD). We exploit this feature to construct a H-NS variant without tryptophan which we term H-NSm (W109F), titrate this variant onto H-NS wildtype (with tryptophan), and monitor the anisotropy changes upon oligomerization of H-NS in the presence of excess H-NSm. Because the DBD is conformationally independent of the dimerization and oligomerization domains, we expect its rotational correlation time to be more sensitive to larger oligomers than monomers or dimers. Accordingly, anisotropy increases only at concentrations beyond 10 μM of H-NSm, establishing that beyond this concentration the protein is well within the oligomerization regime (Figure 2C). This result in turn explains the reason for $c( s )$ distributions of individual peaks shifting to higher values, as the protein concentrations employed falls within the ‘reaction boundary’ conditions of the AUC experiment. The extent of oligomerization decreases with temperature as evidenced by the anisotropy at 50 *μ*M dropping by nearly 27% between 13 and 37°C.

AGADIR (50) predicts variable helical propensities along the length of H-NS with the dimerization and the oligomerization site being weakly and strongly helical, respectively (Figure 2D). In fact, the N-terminal dimerization domain is only marginally stable in solution undergoing an order-disorder transition in the physiological range of temperatures (28). Taken together with the helical nature that is stabilized by local interactions, the structure of H-NS is expected to display strong temperature dependent melting. True to this expectation, the overall helicity from far-UV CD experiments is calculated to be ∼40% at 5°C that drops to 25% at 37°C, the optimal growth temperature of *E. coli* (Figure 2E). This is more clear in the plot of mean residue ellipticity as function of temperature that displays a steep temperature dependence (Figure 2F). The consequence of this observation is that any estimate of oligomerization at low temperature or structural models that are fully folded will not reveal the true physiological oligomerization status of H-NS. Effectively, we provide strong evidence that H-NS not only exhibits heterogeneity in the oligomeric nature (Figure 2G) but also in terms of structure which is intrinsically sensitive to temperature.

### A thermodynamic framework to quantify temperature-dependent populations of oligomeric species

AUC experiments are conventionally performed at 20°C and provide only a glimpse into the nature of oligomeric species and their populations (which are estimated by calculating the area under each of the $c( s )$ distributions). The oligomeric assemblies are destabilized at higher temperatures (Figure 2C) and this is also convoluted by temperature dependent folding-unfolding equilibrium of the monomeric form (Figure 2F). A classic method to dissect the interplay among dimerization, higher-order oligomerization and unfolding involves measuring heat capacity changes with temperature. Heat capacity profiles from differential scanning calorimetry experiments have been at the forefront of calibrating and validating thermodynamic models of folding and oligomerization (51–55). This is because heat capacity is intrinsically related to the underlying partition function; a thermodynamic model can further be compared in a (semi-)quantitative manner with experimental profiles to test the assumptions implicit in modeling.

**Figure 3.**
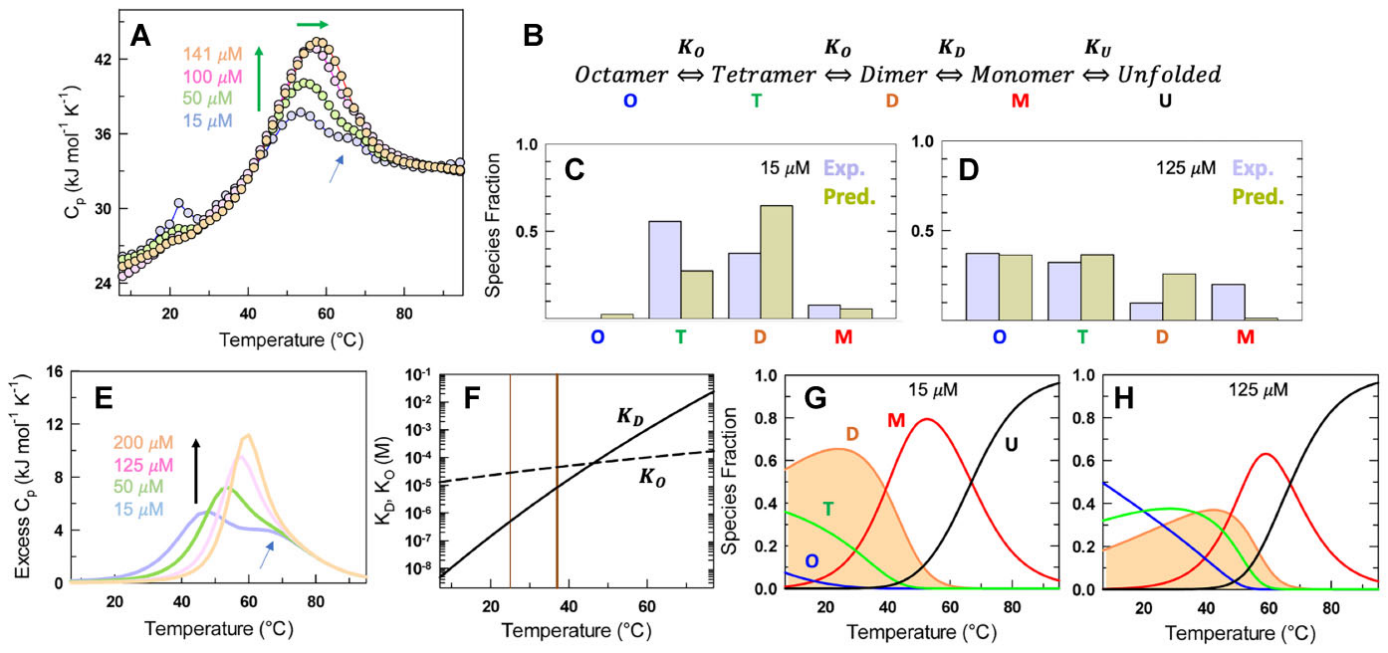
A thermodynamic framework for temperature-dependent H-NS disassembly and unfolding. (**A**) Heat capacity thermograms of H-NS at 150 mM ionic strength, pH 7.0. The green arrows indicate that both the amplitude of the thermogram and the peak temperature increase with protein concentration. The light-blue arrows points to the presence of a second transition at low concentrations which merges with the global transition at higher protein concentrations. The measurement errors in heat capacity are of the order of 0.5 and <0.2 kJ mol^−1^ for lower (<60 μM) and higher protein concentrations (>60 μM), respectively. (**B**) A thermodynamic model for H-NS oligomerization (O), dimerization (D) and unfolding (U). (**C, D**) Comparison of experimental (from AUC experiments) and predicted populations of oligomeric species. (**E**) Simulated heat capacity curves. The light-blue arrow again shows that the model captures the experimental observations faithfully. (**F**) Dependence of ${K}_O$ and ${K}_D$ on temperature. The light and dark brown vertical lines indicate 25 and 37°C, respectively. (**G, H**) Temperature-dependent populations of the different species in the H-NS ensemble.

We therefore generated heat capacity profiles of H-NS at a range of concentrations (Figure 3A). All the profiles are characterized by steep pre-transition regions (5–37°C), indicative of changes in structure or oligomeric nature even at the lowest temperature ranges. Both the apparent peak transition temperatures and the amplitude of the transitions increase with concentration, and this is a consequence of a higher population fraction of oligomeric assemblies at higher concentrations as evidenced from SEC and AUC studies (Figure 2). Two specific observations stand out. First, at 15 *μ*M, two transitions are observable with the stationary points (local maxima) at 52°C and 67°C. Second, a shoulder is visible at ∼67°C for 50 μM protein, but merges with the first transition at the higher concentrations of 100 and 141 μM.

What is the simplest thermodynamic model that explains both AUC and DSC data? The observation of four distinct peaks in $c( s )$ distributions at higher concentrations could mean that there is an equilibrium between monomer, dimer, trimer and a tetramer. This model can be qualitatively ruled out as a fully folded tetramer is expected to have an $s$-value of 2.44 S, while the experimental values span a range which is nearly twice this value. One could invoke a more complex equilibrium involving progressive assembly of monomers to form a continuum of molecular species involving monomer, dimer, trimer etc. Such an expectation is also ruled out as we see only a few discrete peaks in the AUC experiments, and not a broad, continuous $c( s )$ distribution (56). An alternative is to consider a simple thermodynamic framework that assumes a progressive growth in the size of the oligomeric species with two monomers forming a dimer, two dimers coming together to form a tetramer, and two tetramers assembling into an octamer, apart from a folding-unfolding equilibrium associated with the monomer (Figure 3B). Modeling this equilibria requires information on oligomerization constant (${K}_O$), dimerization constant (${K}_D$), unfolding equilibrium constant (${K}_U$), and their respective temperature dependencies (see Methods). The steep pre-transition in DSC precludes fitting the data directly, and we therefore perform a semi-quantitative analysis to capture both the populations in AUC and the relative shape of DSC curves at the experimental concentrations.

The best simultaneous agreement between experimental and simulated monomer-oligomer populations from AUC, and the shape of DSC curves is obtained when the dimerization and oligomerization equilibrium constants exhibit strong and weak temperature dependencies, respectively. The relative populations of octameric species at low and high protein concentrations are captured very well by the model, but with slightly lower accuracy for other oligomeric species (Figure 3C, D). This is not surprising as we employ a minimal model with just eight thermodynamic parameters that does not account for partial unfolding within the oligomer. A more complex mechanism with additional oligomeric species cannot be ruled out but requires the use of more parameters (say, heat capacity change) that inherently makes the predictions less reliable. Despite the minimalistic nature of the model, the predicted DSC curves agree very well with experiments including the observation of two transitions at 15 μM, the shoulder at 50 μM and single sharp transitions for 100 and 141 μM (Figure 3E). Note that both the relative amplitude of the transitions and the shift in apparent melting temperatures are reproduced at a semi-quantitative level.

The resulting equilibrium constant for dimerization is predicted to be significantly stronger than the oligomerization constant by nearly 2 orders of magnitude at 25°C (${K}_D$∼500 nM, ${K}_O$∼30 μM) while they are comparable at 37°C (${K}_D$∼8 *μ*M, ${K}_O$∼45 μM) (Figure 3F). Therefore, it is the dimer that ‘seeds’ the self-association into higher order oligomers, with the latter displaying a much lower stability. This in turn results in partially decoupled transitions in the DSC profile at lower concentrations - the low temperature transition arises due to de-oligomerization (including dimers) while the high temperature transitions originate from the unfolding of the monomeric protein. At higher concentrations, these transitions merge into a single sharp cooperative peak due to the higher fraction of oligomeric species. These differences are better captured in the plots of species fraction as a function of temperature (Figure 3G, H). At 15 μM and at 37°C, the native ensemble is dominated by dimers, monomers and tetramers (in that order; Figure 3G). At 125 μM and 37°C, only minor populations of the monomers are observed with the ensemble populated by mixtures of tetramers, dimers and octamers of H-NS (Figure 3H). The dependence of the shape of heat capacity profile on different parameter combinations is provided in Supplementary Figure S2 in the supporting information, effectively ruling out weak temperature dependence on ${K}_D$, strong temperature dependence on ${K}_O$ and stronger oligomerization than dimerization (${K}_O \leq {K}_D$; Supplementary Table S1).

### Higher osmolarity modulates assembly cooperativity

**Figure 4.**
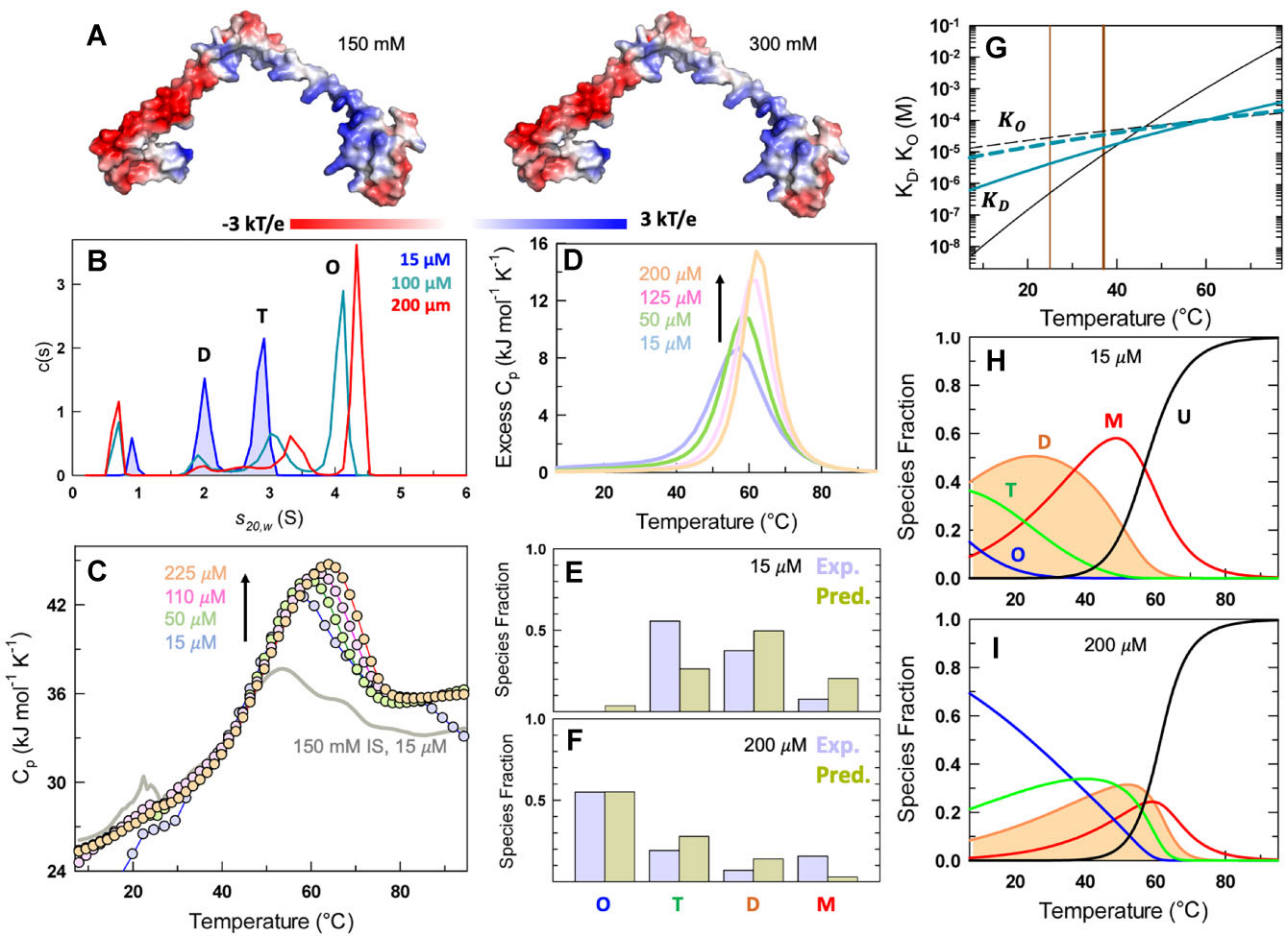
Higher osmolarity modulates cooperativity of unfolding. All experiments are done at 300 mM ionic strength, pH 7.0. (**A**) Electrostatic potential map of H-NS at 150 mM (left) and 300 mM (right). (**B**) Distribution of sedimentation coefficients highlighting the different oligomeric species following the convention in Figure 3. **(C**) Scanning calorimetry profiles as a function of concentration (circles). The data at 150 mM ionic strength and 15 *μ*M protein concentration is shown for reference. (**D–F**) Predicted heat capacity profiles from the thermodynamic model (panel D), and the comparison with AUC derived populations at 20°C (panels E, F). (**G**) Dependence of ${K}_O$ and ${K}_D$ on temperature at 300 mM IS (dark cyan). The dependence at 150 mM is also shown as reference. The light and dark brown vertical lines indicate 25 and 37°C, respectively. (**H, I**) Temperature-dependent populations of the different species in the H-NS ensemble.

H-NS has also been reported to be sensitive to changes in osmolarity, and in fact osmo-sensing is one of the functions attributed to it. H-NS being a non-spherical DNA binding protein necessarily harbors multiple charged residues across its sequence (and not just in the DBD), not only for functional reasons but also to enhance solubility. Accordingly, electrostatic potential calculation reveals a distinct distribution of positive and negative surfaces on the protein, with nearly the entire protein displaying a weaker potential at a higher ionic strength (IS) of 300 mM (Figure 4A). Sequence analysis further suggests that the dimerization site is more hydrophobic than the oligomerization site (Figure 1A), with the residues involved in oligomerization being primarily charged and stabilizing the assembly. Specifically, K57–D68* (* indicating that the residue is located in the other protomer), R54–E74* and K83–D71* ionic interactions (19) dominate the oligomer interface. However, the R15-E24*/E27* ionic interactions (24) and leucine-zipper-like interactions (57) between L25 and V36*, and L29 and L32* stabilize the dimerization interface. Given that dimerization is critical for oligomerization as inferred from the experiments above, we hypothesize that H-NS should populate an altered distribution of oligomeric species at 300 mM ionic strength.

To our surprise, AUC experiments reveal similar distributions of tetramer, dimer and monomer with no octameric species at low concentrations and at 300 mM IS (Figure 4B). On increasing the protein concentration, however, we notice a notable increase in fraction of octameric species with minimal populations of other oligomeric states. This increase in apparent cooperativity at higher osmolarity is visually evident in the heat capacity profiles that display only a single transition in the range of protein concentrations explored (15–225 μM; Figure 4C). The 15 μM profile is quite distinct at 300 mM IS with a higher heat capacity peak and a single cooperative transition. The merging of the two transitions (observable at 15 μM, 150 mM IS) hints that the oligomerization and dimerization equilibrium constant are similar at 300 mM ionic strength. Moreover, the solubility of H-NS is much higher at 300 mM IS (and hence the higher protein concentrations explored) signaling a non-trivial effect of solvation and charge-screening on the structural and self-associative properties of the protein. The modeled heat capacity profiles that simultaneously predict oligomer species fraction in a semi-quantitative manner are shown in Figure 4D–F. The extracted dimerization equilibrium constant is shifted upwards that is indicative of weaker dimerization, and the oligomerization equilibrium constant is predicted to be slightly stronger (Figure 4G; Supplementary Table S2). The smaller difference between the two equilibrium constants conspire to result in a single cooperative transition in DSC, unlike the 150 mM profiles that display decoupled transitions. The species fractions display a similar trend to the 150 mM IS data, with the primary difference being that the unfolding curves are noticeably sharper (Figure 4H, I).

### A phosphomimetic mutation eliminates oligomerization but preserves dimerization

**Figure 5.**
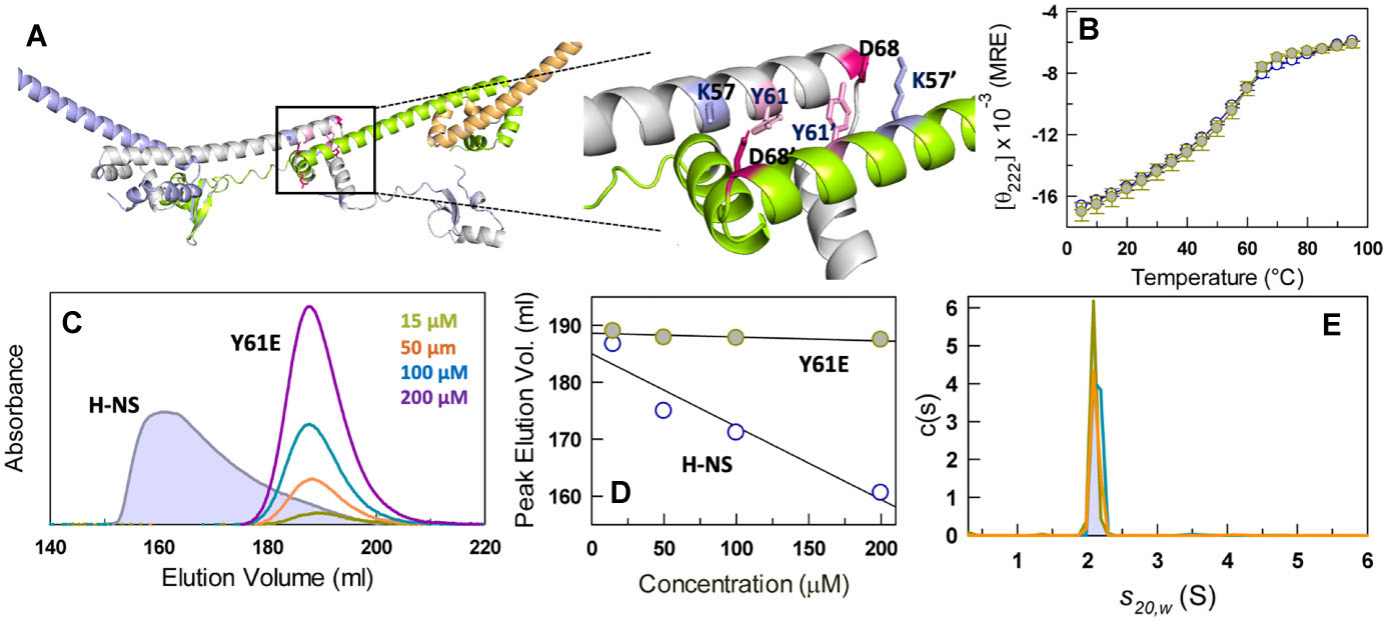
Phosphomimetic mutation-induced switch in ensemble polydispersity. **(A)** The oligomer interface of a tetramer structural model (left) which is zoomed-in (right) to highlight the local environment around Y61, both within and across the protomer (the residues of the second protomer are marked with an apostrophe). **(B)** Comparison of the mean thermal unfolding curves monitored by far-UV CD at 222 nm for H-NS (open circles) and the Y61E mutant (filled circles). Errors reported are SEM from 3 replicates. **(C)** Y61E elutes as a single sharp peak irrespective of protein concentration at 300 mM ionic strength at 25°C. The elution profile of H-NS at 200 *μ*M protein concentration is shown as a reference. **(D)** The peak elution volume as a function of protein concentration. **(E)** Distribution of sedimentation coefficients reveal a single sharp distribution corresponding to a dimeric protein. Dark cyan and orange represent the distributions at 125 *μ*M protein, 150 mM ionic strength and 200 *μ*M protein, 300 mM ionic strength, respectively.

One of the primary predictions of the thermodynamic model is that dimerization is much stronger than higher-order oligomerization. Here, we exploit the presence of a phosphorylation site, Y61 (tyrosine 61), identified by mass spectrometry (58) and introduce a phosphomimetic mutation, Y61E, to test this expectation. Structurally, Y61 is proximal to K57 in the same chain that makes a strong salt-bridge with a D68’ in the chain adjacent to it (Figure 5A). A Y61E mutation is expected to strongly destabilize this interaction and weaken higher-order oligomerization. The far-UV CD unfolding curve of the mutant Y61E however resembles that of the wild-type with little or no change in the signal magnitude or unfolding cooperativity (Figure 5B; *vide infra*).

SEC, though, reveals a dramatic shift in the elution profile with Y61E eluting nearly 30 ml later compared to the wild-type H-NS at 200 *μ*M (Figure 5C). Furthermore, the peak elution volume displays very little dependence on the protein concentration (Figure 5C, D). Consistent with these results, AUC reveals a single $s$-value distribution centered around 2.1 S and remarkably, independent of protein concentration (15 – 200 *μ*M) or ionic strength (150 and 300 mM; Figure 5E, Supplementary Figure S3). As the mutation strongly destabilizes oligomerization, this peak corresponds to that of a H-NS dimer and validates our earlier assignment (Figure 2B). Eliminating oligomerization thus transforms the native ensemble of the mutant to be fully monodisperse. One other observation that stands out is the absence of a peak corresponding to the monomer (at ∼1.1 S) in the Y61E $s$-value distribution (compare Figure 2B or 4B with Figure 5E). This effectively means that oligomerization intrinsically weakens the dimerization promoting the population of monomeric species, despite the two sub-domains being > 40 Å apart. In the absence of oligomerization (as in the Y61E mutant), this allosteric or long-distance effect is not present and hence the dimer is observed to be the only stable species.

### Cross-talk between oligomerization and dimerization sites

**Figure 6.**
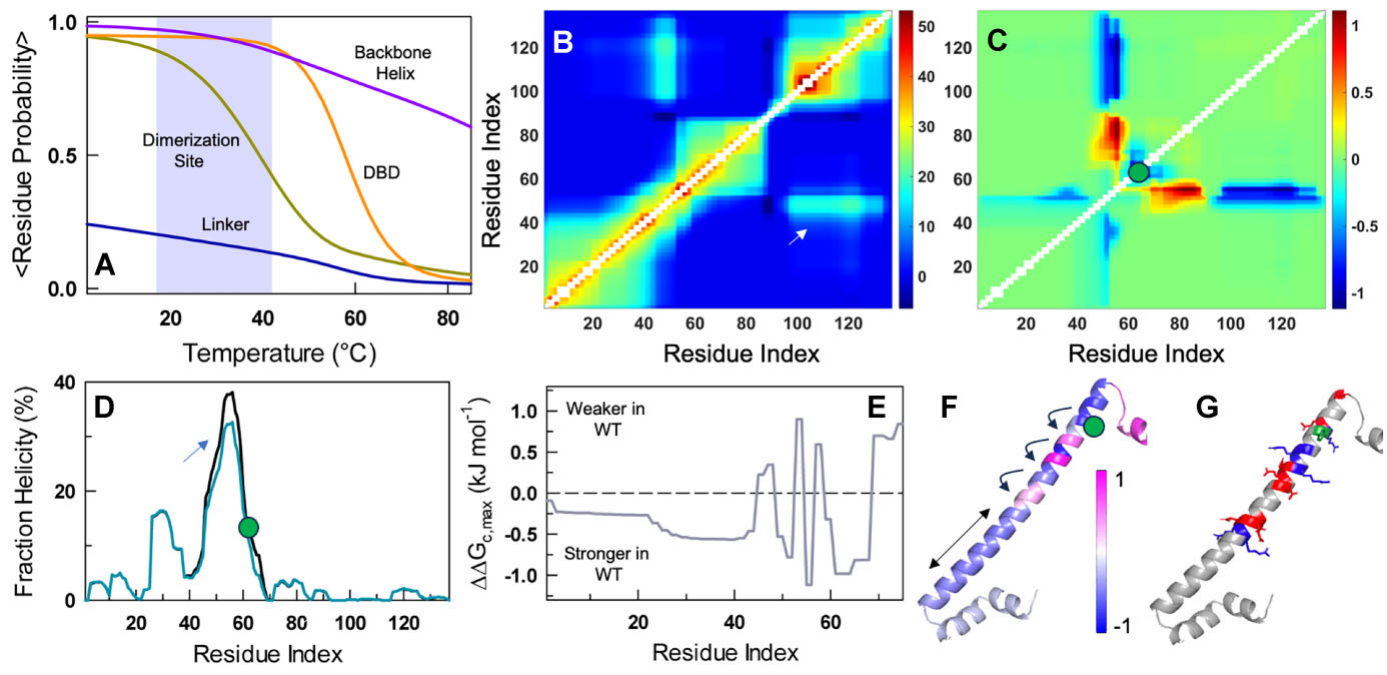
Long-range connectivity between dimerization and oligomerization sites. **(A)** Thermal unfolding curves of H-NS structural elements. The shaded region represents a temperature range in which the dimerization site displays maximal changes and matches the physiological range. Note that the backbone helix is quite stable owing to its high intrinsic helical propensity. **(B)** Effective thermodynamic coupling free energy ($\Delta {G}_c$) between every residue and every other residue at 25°C. The color bar indicates the range in kJ mol^−1^. Note that the backbone helix appears to be coupled to the DBD as both are fully folded at this temperature. **(C)** The difference in coupling free energy (in kJ mol^−1^) between the wild-type H-NS and the Y61E mutant, showcases the long-range effects of mutation on the coupling free energy. The position of Y61 is shown as a green circle. **(D)** AGADIR predictions of helical propensity for H-NS (black) and the Y61E mutant (dark cyan). The position of Y61 is shown as a green circle to highlight the fact that while there is no dramatic change in helical propensity at the site of mutation, residues farther from this site are perturbed due to the helical nature (see main text). **(E)** Differences in maximal coupling free energy between the WT and the mutant as a function of residue index spanning the dimerization and oligomerization sites. Negative sign represents regions that are more strongly coupled in the WT compared to the mutant and *vice versa*. **(F)** The data in panel E mapped on to the structure. The curved arrows point to a domino-like effect of mutations that spread all the way to the dimerization site (straight arrow). **(G)** Cartoon representing the series of charged residues around the phosphorylation site in green. Acidic and basic residues are represented in red and blue, respectively. The linker region and DBD are not shown for clarity.

The question is then, are the two sites connected thermodynamically? In other words, is there a cross-talk between the two sites that is modulated by phosphorylation, or as in this study by the phosphomimetic Y61E mutation? To probe this, we employ the block version of the statistical mechanical Wako-Saitô-Muñoz-Eaton (WSME) model (42) that considers an ensemble of > 1.3 million substates corresponding to the monomeric version of H-NS (Methods). Since the stability of H-NS is concentration dependent, the true energy scale is difficult to ascertain. However, the DNA binding domain (DBD) behaves as an independent unit displaying a concentration-independent melting temperature of 57°C (Supplementary Figure S4). We therefore calibrated the model to reproduce the ${T}_m$ of the DBD by adjusting a single parameter, the van der Waals interaction energy per native contact. The resulting thermal unfolding curves of the monomeric variant reveal insights into the distribution of stability patterns: the stability of linker < dimerization site < DBD < backbone-helix including the oligomerization domain (Figure 6A). The high stability of the backbone-helix is in very good agreement with the large helical propensity for the same predicted by AGADIR (Figure 2D).

The high stability and continuous nature of the long backbone-helix could serve as a ‘information conduit’ connecting the oligomerization and dimerization sites. We test this by calculating the parameter effective coupling free energy (${\mathrm{\Delta }}{G}_c$), a pairwise term, that reports on the coupling between one site to another through direct contacts, second- and third-shell effects and through redistribution of populations in the native ensemble (43). Stronger thermodynamic coupling signals that the folding status of one residue is intimately connected to another and *vice versa*, and thus reports on the allosteric outcomes. The effective coupling free energy map resembles that expected for a helical system dominated by strong local coupling (i.e. close in sequence and hence stronger coupling along the main diagonal; Figure 6B). An exception is the region 48–58 that forms a part of the backbone helix whose conformational status is coupled to the DBD, simply because both are folded at lower temperatures, and hence not considered further.

The difference matrix, calculated as ${\mathrm{\Delta }}{G}_{c,Y61E} - {\mathrm{\Delta }}{G}_{c,HNS}\ ( {\Delta \Delta {G}_c} )$, reports on the extent to which segments of the protein are more (or less) coupled in the mutant compared to the WT (Figure 6C). The mutational effect is not localized to position 61, but spreads around the structure, strongly affecting the residue stretch 48–58. In fact, independent predictions by the program AGADIR reveals that Y61E reduces helical propensity in the regions adjacent to it through a complex network of $i - i + 4/5$ side-chain interactions involving R56, K57, which in turn modulates interactions with E53 located N-terminal to it (Figure 6D). These long-range effects are evident when the maximum effect on the differences in coupling free energy ($\Delta \Delta {G}_{c,max}$) are plotted as a function of sequence index (Figure 6E). Residues 1–45 encompassing the oligomerization site, and 61–70 are more strongly coupled in the WT compared to the mutant. Mapping the free-energies on to the structure, we find a domino-like effect of the Y61E mutation that (de-)stabilizes regions around it through adjacent $i - i + 4/5$ interactions, thus subtly determining the conformational status of the oligomerization site (Figure 6F, G). Finally, though the dimerization site of the monomeric protein is weakly stable, it is expected to be stabilized on dimerization. Upon phosphorylation (or phosphomimetic mutation), the thermodynamic connectivity between the two sites acts to enhance the stability of the dimeric species much more effectively, thus eliminating the monomeric state (compare Figure 2B or 4B with Figure 5E; also *vide infra*).

### Oligomerization is a pre-requisite for strong DNA binding

**Figure 7.**
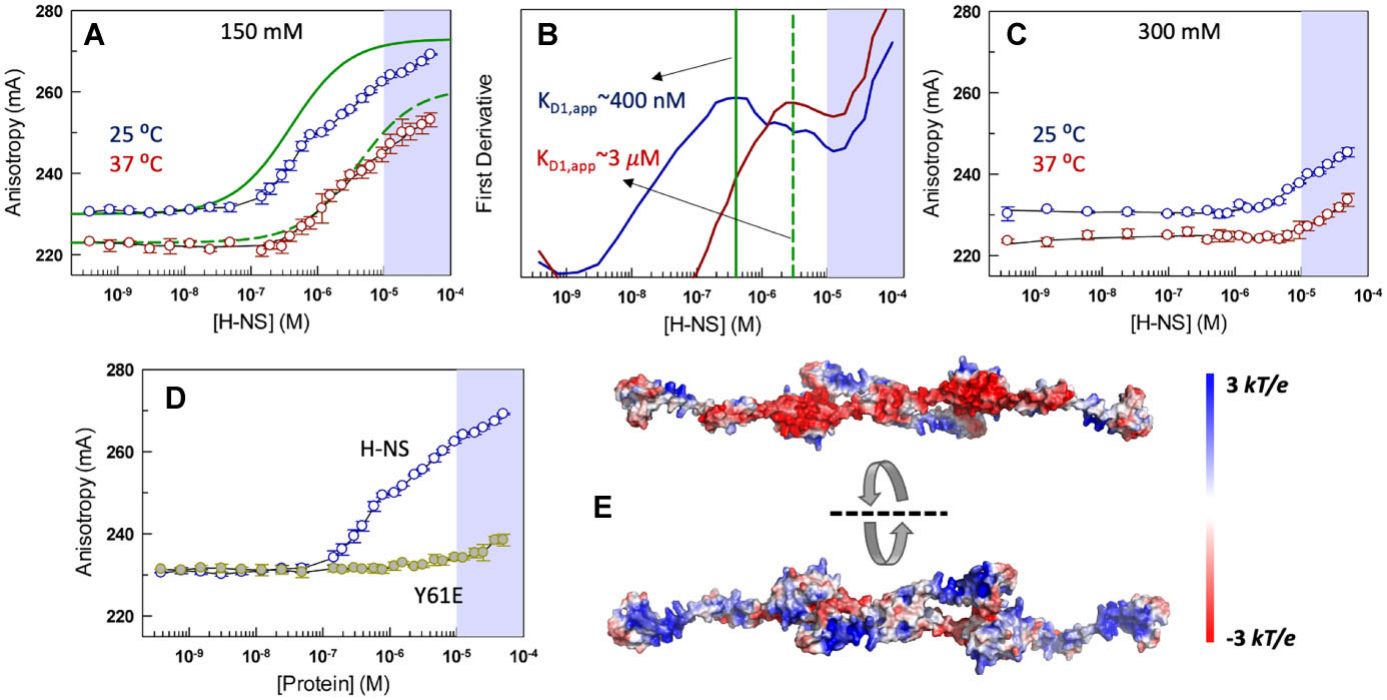
Higher-order oligomerization of H-NS is necessary for strong DNA-binding. All errors reported are SEM from 3 replicates. (**A**) Anisotropy of Cy3-labeled 100-bp DNA as a function of H-NS concentration (circles). Green curves are expectations based on a 1:1 binding equilibrium model. The shaded region highlights non-specific binding. (**B**) First derivatives of the data shown in panel A and the corresponding apparent dissociation constants. (**C**) Same as panel A but for 300 mM ionic strength condition. Black curves are shown to guide the eye. (**D**) Y61E binds only weakly to DNA even at 150 mM ionic strength. (**E**) Electrostatic potential map of a H-NS tetramer model at 150 mM ionic strength and 25°C.

Here, we perform binding assays by titrating H-NS onto a Cy3-labeled 100-bp DNA to estimate the apparent dissociation constants (${K}_{D,app}$). The anisotropy remains constant at low protein concentrations and at 150 mM IS, while it starts rising beyond 20 nM at 25°C, and after 1 *μ*M at 37°C (Figure 7A). Neither of the binding isotherms reach saturation and increase in magnitude on addition of protein even up till 100 μM. This is better observable when comparing the experimental data to the expectations from a classical 1:1 binding equilibrium model that saturates within two orders of magnitude in protein concentration. There is no simple avenue available to quantify or model this binding isotherm due to the polydisperse nature of H-NS. Since H-NS binds non-specifically to DNA, every duplex in solution can not only be bound at different sites along the sequence, but also through multiple combinations of oligomeric states. Furthermore, the oligomeric status can itself be modulated in the presence of DNA. Therefore, we took a first derivative of the binding isotherm to identify inflection points that report on the ${K}_{D,app}$. The ${K}_{D1,app}$ is estimated to be ∼400 nM at 25°C that weakens to ∼3 μM at 37°C, indicative of weaker binding at the higher temperature (Figure 7B; *p*-value = 0.012). A second binding regime is observed at both temperatures beyond 10 μM since the anisotropy signal does not saturate. H-NS thus appears to bind via two ‘modes’, a temperature-dependent specific mode (${K}_{D1,app}$) and a temperature-independent non-specific mode (shaded region). Higher ionic strength eliminates the specific mode in its entirety and at both 25 and 37°C, as observed by the lack of signal increase at H-NS concentrations lower than 10 μM (Figure 7C). The non-specific binding mode still persists even at higher ionic strength highlighting that the two different binding modes potentially originate from a requirement to bind at different ionic strength or temperature conditions. An alternate and simpler interpretation is that the ${K}_{D,app}$ of the specific binding mode is shifted to higher concentrations, which would also explain the weaker binding observed at higher ionic strength and temperature. However, these interpretations need to be verified via additional experiments probing the structural organization in the presence of DNA.

One of the enduring questions in studies related to H-NS is the extent to which oligomerization drives DNA binding. We answer this by measuring the anisotropy as a function of Y61E protein concentration. To our surprise, we observe that the binding to DNA is dramatically weakened in the mutant even at 25°C and 150 mM ionic strength (as observed via the minimal change in anisotropy amplitude in the same range of concentrations; Figure 7D). Oligomerization, in fact, promotes the build-up of a significantly large positive electrostatic potential surface on one face of an H-NS assembly, and a concomitant negative electrostatic potential on the opposite face (Figure 7E). Based on these experimental and structure-based observations, we hypothesize that the effective combination of oligomerization and favorable electrostatic potential drives the DNA binding of H-NS in a sequence non-specific manner. In the dimer (i.e. the Y61E variant), the surface potential is significantly reduced and hence binding is observed only at very high concentrations.

### Co-repressor binding, allostery and enhanced DNA binding

**Figure 8.**
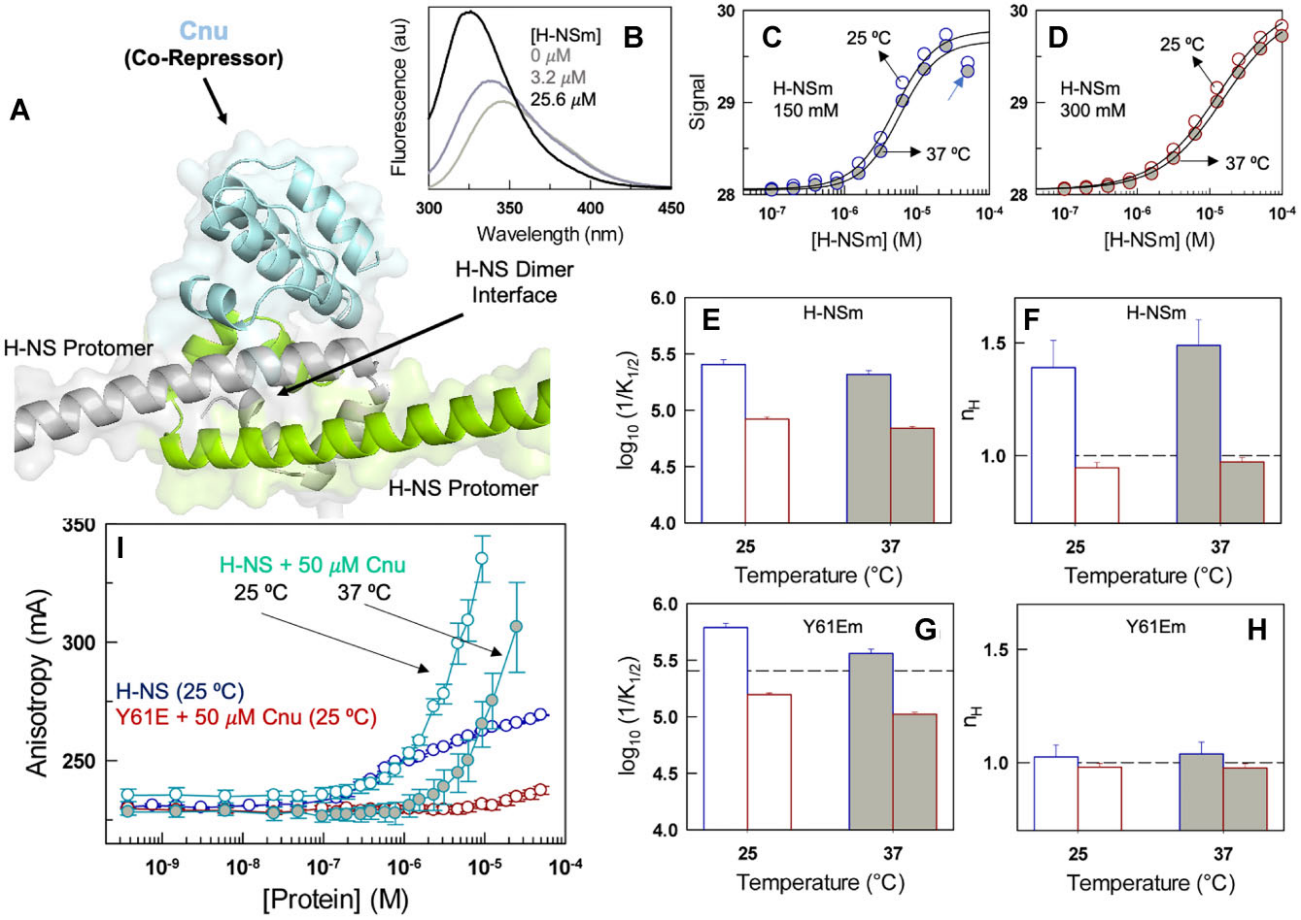
Non-trivial outcomes of co-repressor binding. (**A**) Structural model of Cnu bound H-NS dimer. (**B**) Titration of H-NSm variant (W109F H-NS) on to 2 μM Cnu that harbors a tryptophan (W67). Both the fluorescence emission maximum and intensity of W67 vary at increasing concentrations of H-NSm. (**C, D**) Intensity-averaged wavenumber (the ordinate; see Methods) as a function of H-NSm concentration at 150 mM (panel C; blue) and 300 mM (panel D; dark red). Empty and filled circles represent 25 and 37°C, respectively. The cyan arrow in panel C points to a roll-over in the data which is not observable in any of the other conditions or in the mutant. (**E, F**) The apparent binding dissociation constant (${K}_{1/2}$; panel E) and the Hill-coefficient (${n}_H$; panel F) extracted from the data in panels C and D following the same color code. (**G, H**) Same as panels E and F but for the titration of Y61Em (Y61E/W109F H-NS) on Cnu. The error bars shown in panels E–H report on the 68% confidence interval of the parameters upon analyzing the data with the Hill equation. (**I**) Anisotropy of Cy3-labeled 100-bp DNA for the different variants and conditions indicated. Note that the shape of anisotropy dependence changes dramatically for H-NS in the presence of 50 μM Cnu. All errors reported in this panel are SEM from 3 replicates.

The condensation of H-NS on DNA and hence the bacterial stress response is additionally regulated by the Hha-family of co-repressors. One of the Hha-family members, YmoA, binds H-NS at the dimerization site forming higher-order polydisperse assemblies including YmoA:(H-NS)_2_ and (YmoA)_2_:(H-NS)_2_ complexes (studied on a truncated version of H-NS harboring only the first 64 residues) (8). We have previously shown that another Hha family member, Cnu, modulates its structure, stability, ensemble dimensions and dynamics in response to changes in osmolarity and temperature (28,59). AlphaFold-ColabFold predicted structural model of Cnu:(H-NS)_2_ complex reveals a similar architecture to YmoA:(H-NS)_2_ with a Cα-RMSD of 0.6 Å (Figure 8A). The complex is characterized by a ‘staple-like’ arrangement, wherein Cnu locks and stabilizes the H-NS dimerization site via a series of charge–charge and cation–π interactions. We employ this system to answer three specific questions: what is the apparent binding affinity of Cnu to full length H-NS, and its dependence on ionic strength? If the dimerization and oligomerization sites are thermodynamically connected, then is the binding to Cnu perturbed by the phosphomimetic mutation? To what extent does Cnu enhance the binding of H-NS to DNA?

Cnu harbors a sole tryptophan (W67) which gets buried fully at the dimerization interface and hence serves as a good probe for H-NS binding (28). As before (Figure 2C), we employed the H-NSm variant (W109F H-NS) to enable monitoring the fluorescence solely from Cnu. Binding promotes blue-shifts in the fluorescence emission maximum of W67 in Cnu in addition to intensity changes (Figure 8B); these two observables are combined into an intensity-averaged wavenumber enabling the monitoring of changes in W67 fluorescence properties at increasing concentrations of H-NSm (Methods). The resulting binding isotherms cannot be analyzed with a simple 1:1 or 1:2 binding equilibrium. This is because the H-NS native ensemble is not composed of a single-species, and different numbers of Cnu could potentially bind to each of the oligomeric species. Furthermore, the degree of oligomerization of H-NS also varies with concentration making it challenging to quantitatively model this feature. We therefore resort to the Hill equation to calculate the fraction bound as


\begin{equation*}f = \frac{{{{\left[ {HNS} \right]}}^{{n}_H}}}{{K_{1/2}^{{n}_H} + {{\left[ {HNS} \right]}}^{{n}_H}}}\end{equation*}

where ${K}_{1/2}$ is the midpoint of binding or the apparent dissociation constant, and the Hill-coefficient${\mathrm{\ }}{n}_H$ represents the cooperativity of binding. As we cannot extract stoichiometries from such a fit for the reasons discussed above, we employ the Hill coefficient to merely highlight differences across conditions, if any.

Temperature modulates the Cnu:H-NSm complex assembly only marginally (slightly right shifted isotherms in Figure 8C, D), but the ionic strength change from 150 to 300 mM destabilizes the complex considerably (${K}_{1/2}$ of 4 ± 0.4 μM and 12 ± 0.5 μM at 150 and 300 mM, respectively, at 25°C; Figure 8E). Importantly, the binding isotherm is sharper than that expected for a 1:1 binding equilibrium at 150 mM resulting in a Hill coefficient of 1.39 ± 0.12 at 25°C which increases to 1.49 ± 0.11 at 37°C (Figure 8F). At H-NS concentrations beyond 20 *μ*M, the signal starts decreasing, signaling enhanced homo-oligomerization of H-NS (as expected from the data in Figure 2) at the expense of hetero-oligomerization with Cnu. In fact, the dissociation constant for H-NS oligomerization (${K}_O$ of 30 μM at 25°C) and ${K}_{1/2}$ of Cnu binding (4 μM at 25°C) are within an order of magnitude under these conditions. Hill-coefficients greater than one suggests that the ensemble is composed of multiple oligomeric species in solution with different stoichiometries, i.e. Cnu:(H-NS)_2_, (Cnu)_2_:(H-NS)_2_, (Cnu)_2_:(H-NS)_4_ etc. Remarkably, at 300 mM ionic strength, the curves are well described by a Hill coefficient of one suggesting that the species diversity in the ensemble has reduced considerably (Figure 8F).

AUC experiments and statistical modeling revealed distinct connectivity between dimerization and oligomerization. If this is indeed the case, then one expects that Cnu binding to the H-NS dimerization site would be perturbed in the Y61E mutant (that forms only dimers). True to this, we observe that Cnu indeed binds stronger in the Y61Em version of H-NS - ${K}_{1/2}$ of 1.6 ± 0.1 μM for Y61Em versus 4 ± 0.4 μM for H-NSm (Figure 8G, Supplementary Figure S5), translating to a coupling free energy of 2.3 kJ mol^−1^ which is within the range expected from the statistical model (Figure 6). The ensemble diversity is proportionately reduced in the Y61E mutant as evidenced from the Hill coefficients that span the range 0.98 – 1.04 under both temperature and ionic strength conditions (Figure 8H). Interestingly, the roll-over in the signal at higher concentrations of H-NS for the 150 mM conditions (Figure 8C) is completely absent for the Y61E mutant (Supplementary Figure S5), confirming that this unique observation is indeed a consequence of higher-order self-oligomerization.

Cnu by itself does not bind DNA, and the binding equilibrium of H-NS to DNA is only minimally altered in the presence of 5 *μ*M Cnu (Supplementary Figure S6). However, on increasing the concentration to 50 μM, the binding isotherm is observed to be atypical with a dramatic enhancement in the anisotropy at 25°C. Beyond 5 μM H-NS the solution of Cnu:H-NS: DNA turns cloudy with a proportionate increase in light scattering at 350 nm (Supplementary Figure S7). A similar increase in anisotropy is observed at 37°C but shifted to higher H-NS concentrations (Figure 8I). We speculate that this is potentially a sign of bridging of DNA by H-NS, wherein large H-NS oligomers are sandwiched between two DNA duplexes, reported in earlier studies (11). This inference is further supported by the lack of increase in anisotropy for the Y61E mutant in the presence of 50 μM Cnu (dark red in Figure 8I).

## Discussion

H-NS and H-NS family of proteins have been at the forefront of understanding bacterial stress-response mechanisms including silencing of foreign DNA and genomic compaction. However, the precise stoichiometry of H-NS assembly has been an open question, because of the challenges inherent to characterizing oligomeric assemblies that do not exhibit a single fixed stoichiometry (60). In this work, we generate a self-consistent picture of the H-NS oligomerization landscape as a function of temperature, osmolarity and protein concentration, apart from insights into hetero-oligomerization in the presence of DNA and the co-repressor Cnu.

The H-NS native ensemble is composed of multiple but discrete oligomeric species including a mixture of monomers, dimers, tetramers and octamers (Figure 2). The $s$-values from AUC are an effective representation of oligomeric nature, structural differences (across conditions), and degree of partial melting or unfolding, apart from confounding factors arising from exchange between species within the time scale of experiments. We therefore performed a detailed analysis of changes in heat capacity that faithfully report on the thermodynamics of assembly and disassembly (Figure 3). We find that the oligomeric nature is determined by an interplay between oligomerization and dimerization constants. At 150 mM ionic strength (unstressed conditions), the dimerization constant is much stronger than the oligomerization constant leading to decoupled transitions in the DSC thermograms. DLS experiments on an H-NS ortholog from *Salmonella typhimurium* report on the possibility of an assembly of 4–8 dimers at 500 μM concentration, with the maximal hydrodynamic radius ${R}_h$ ranging between 12–20 nm for 125–500 μM protein concentration range (29). With the maximal *in vivo* concentration of H-NS being ∼130 μM, (61) the octameric assembly is an upper limit under these conditions, though macromolecular crowding enhances oligomerization (30). We stress here that other oligomeric species exist in equilibrium with the large octameric assembly as can be directly inferred from AUC experiments. In fact, the oligomeric species distribution in H-NS falls in the range expected for bacterial assemblies identified in a large-scale study involving 17 proteins and through a range of experimental methods (60).

The population of higher-order assemblies decrease with temperature, exchanging with oligomers of lower stoichiometry and eventually with the monomer which unfolds at higher temperatures. The oligomerization landscape of H-NS is therefore intrinsically sensitive to temperature which could explain the diverse reports on the extent of oligomerization. At 300 mM ionic strength, the dimerization is weakened, and the oligomerization is marginally strengthened (relative to 150 mM) resulting in a sharp cooperative melting of oligomers (Figure 4). The difference in cooperativity is suggestive of changes in the architecture of H-NS oligomer arising from a combination of modulation of electrostatic interactions and solvation differences. In fact, studies on the paralog MvaT from *Pseudomonas aeruginosa* reveal salt dependent changes in the conformation of dimeric assemblies (18) in line with the observations presented in the current work. One important aspect that is not addressed in the current work is the role of the more physiological potassium and glutamate ions (62) in modulating protein-protein and protein–DNA interactions. Similarly, other relevant molecules such as polyphosphates (63) and ATP (64) could modulate the different equilibria, contributing to conformational responses tailored to different conditions.

A lingering question in the study of oligomeric assemblies is the mechanism through which such large oligomers are disassembled to reversibly regulate functional outcomes. We find that a previously identified phosphorylation site, (58) Y61, when mutated to glutamate (i.e. a phosphomimetic mutation) switches the highly polydisperse WT ensemble to a monodisperse ensemble populated solely by dimers (Figure 5). Since the primary function of H-NS family members is to control gene expression, disassembly alone is not sufficient but it should be coupled to altered DNA binding. (35,37) We indeed find that DNA binding is ultimately linked to the extent of oligomerization – the WT protein binds DNA via two different ‘modes’ (specific and non-specific, likely) while the Y61E mutant that exists solely as a dimer is unable to bind DNA strongly (Figure 7).

**Figure 9.**
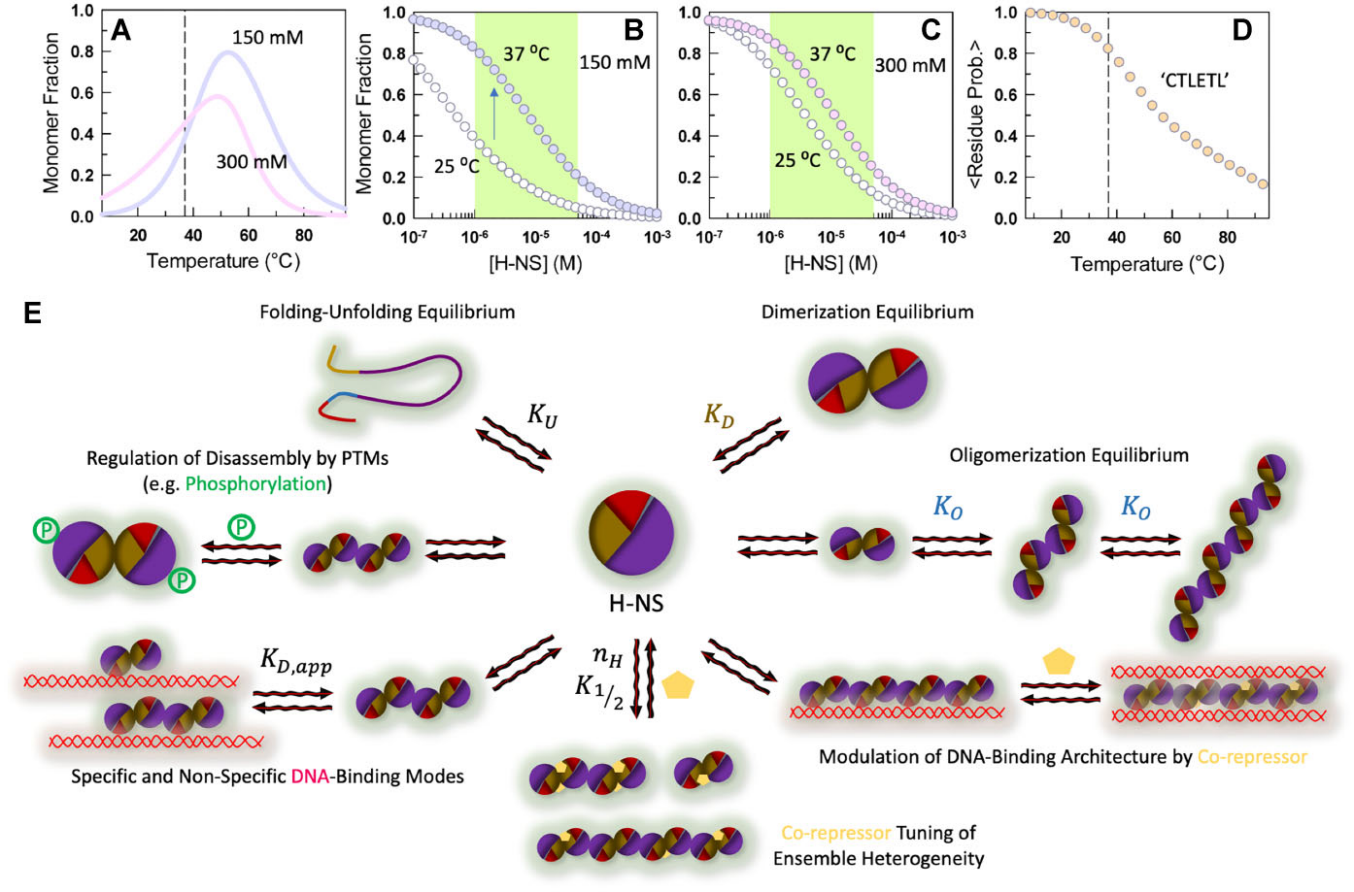
A hierarchical multi-level response of the H-NS ensemble to changing ambient conditions. (**A**) Temperature-dependent monomer populations as predicted by the thermodynamic model at 15 *μ*M and at the indicated ionic strength (from Figures 3G, 4H). The vertical dashed line indicates 37°C. (**B, C**) Monomer concentration as a function of total H-NS concentration (abscissa). The shaded region represents a regime where the differences in cooperativity between 150 and 300 mM ionic strength are more apparent. (**D**) The mean residue folding probability of the H-NS motif ‘CTLETL’ (that is recognized by Lon protease) as a function of temperature as predicted by the bWSME model. The vertical dashed line indicates 37°C. (**E**) A schematic highlighting the different states of the H-NS monomer that act to control gene expression, besides concentration dependent effects (panel B, C). Note that we do not directly observe bridged DNA duplexes in the current work.

The weaker binding of H-NS to DNA at higher temperatures (say, 37°C compared to 25°C) is therefore not driven merely by differences in thermal energy, but also through reduced population of higher-order assemblies, as oligomerization is strongly temperature-dependent in the range 20–40°C. This feature potentially enables the switch between repression and activation depending upon the ambient temperature. Since the body temperature of a mammalian host is 37°C, enterobacteria will be able to constitutively turn on gene expression as the extent of DNA binding by H-NS will be dramatically weakened due to reduced oligomerization. Recent experiments reveal that enhanced degradation of H-NS upon the exposure of specific residues to the Lon protease (the ‘CTLETL’ motif in the N-terminal dimerization site) leads to a successful colonization of the mammalian gut (23). This is possibly because the monomer population increases at 37°C (a sign of successful infection) relative to 25°C irrespective of the ionic strength (Figure 9A, as predicted by the thermodynamic model). However, because H-NS is an oligomeric protein, a decrease in overall concentration increases the monomer concentration even further at the expense of higher-order assemblies (i.e. as [H-NS] approaches ${K}_O$ or ${K}_D$ or lower), with the effect being more significant at 150 mM ionic strength compared to 300 mM IS (Figure 9B, C). The folded status of the ‘CTLETL’ motif also decreases with temperature (Figure 9D, as predicted by the bWSME model). Thus, a switch in temperature upon successful infection will set up a chain-reaction at different levels leading to degradation of H-NS.

The Hha-family of co-repressors add an additional layer to the complex regulation mechanisms omnipresent in bacteria. They not only bind to the H-NS family of proteins at the dimerization site, but also display their own thermo-osmo-pH sensitive behavior (8,28,59,65,66). We identify that Cnu, a member of the Hha family in *E. coli*, binds to both H-NS WT and its Y61E mutant, but with a stronger binding affinity for the latter. The difference, though small, demonstrates the presence of long-range thermodynamic connectivity between the dimerization and oligomerization sites. In other words, the architecture of the (H-NS)_2_:Cnu complex is not identical to that of (H-NS)_4_:Cnu and similar higher-order hetero-oligomeric assemblies. The Cnu-stapled assembly of H-NS binds DNA in a distinct manner contributing to a large enhancement in anisotropy within a narrow dynamic range of H-NS concentrations (Figure 8I). In this regard, two different mechanisms of DNA binding by H-NS have been proposed from prior studies – one in which H-NS coats the DNA lattice linearly and another in which it bridges two DNA duplexes (11). In addition, perturbations (say, temperature) cause structural changes in the oligomerization domain, leading to a switch from ‘closed’ to ‘open’ conformations aided by interactions between N- and C-terminal domains (29). As the sharp change in anisotropy originates from a dramatic reduction in the rotational correlation time of the molecular assembly involving DNA, this *could* signal a transition from a linearly coated array of H-NS on DNA to bridged DNA duplexes. Curiously, the AlphaFold-ColabFold predicted octameric assembly models exhibit a pattern wherein adjacent DBDs point in the opposite direction (primed to bind two DNA-duplexes; Figure 2G), the relative position of which could be controlled by the extent of Cnu binding (which is in turn determined by the concentration). A caveat, however, is that the structural model of the octameric assembly has a lower confidence score from the AlphaFold-ColabFold interface (Supplementary Figure S1) compared to other assembly modes. Despite this, it is evident that the co-repressor could therefore play a vital role in not just regulating the oligomeric nature of the H-NS assembly, but also its DNA binding properties.

In summary, we showcase the presence of a multi-level response to changing ambient conditions in the enterobacterial sensory protein H-NS (Figure 9E). This arises from an intricate interplay between and across multiple thermodynamic variables including modulation of the folding-unfolding equilibrium (level 1, ${K}_U$), dimerization equilibrium (level 2, ${K}_D$), higher-order oligomerization or self-assembly (level 3, ${K}_O$), post-translational modification extent and identity (level 4, studied in the current work through phosphomimetic mutation), specific and non-specific DNA-binding modes (level 5, ${K}_{D,app}$), co-repressor binding affinity and cooperativity (level 6, ${K}_{1/2}$ and ${n}_H$), and finally the binding mode of co-repressor bound H-NS to DNA (level 7). Each of these thermodynamic variables could be further tuned by temperature, osmolarity (including different salt types), pH, and macromolecular crowding, effectively determining the cellular fate via repression and de-repression of genes.

## Supplementary Material

gkae090_Supplemental_File

## Data Availability

The data underlying this article are available in the article and in the online supplementary material.
