# Supplementary material for: Molecular and thermodynamic determinants of self-assembly and hetero-oligomerization in the enterobacterial thermo-osmo-regulatory protein H-NS
Source: Nucleic Acids Res. 2024 Feb 10;52(5):2157–73. doi: 10.1093/nar/gkae090 (PMC10954469; doi:10.1093/nar/gkae090)
Supplement: gkae090_Supplemental_File [file gkae090_supplemental_file.pdf]

Supplemental Information

Molecular and Thermodynamic Determinants of Self-Assembly and Hetero-Oligomerization in the Enterobacterial Thermo-Osmo-Regulatory Protein H-NS

Bincy Lukose,[1] Takahiro Maruno,[2] Mohammed A. Faidh,[1]

Susumu Uchiyama[2] & Athi N. Naganathan*[,1]*

[1]Department of Biotechnology, Bhupat & Jyoti Mehta School of Biosciences, Indian Institute of Technology Madras, Chennai 600036, India.

[2]Department of Biotechnology, Osaka University, Japan.

AUTHOR INFORMATION

Corresponding Authors

e-mail: athi@iitm.ac.in, suchi@bio.eng.osaka-u.ac.jp

Phone: +91-44-2257 4140

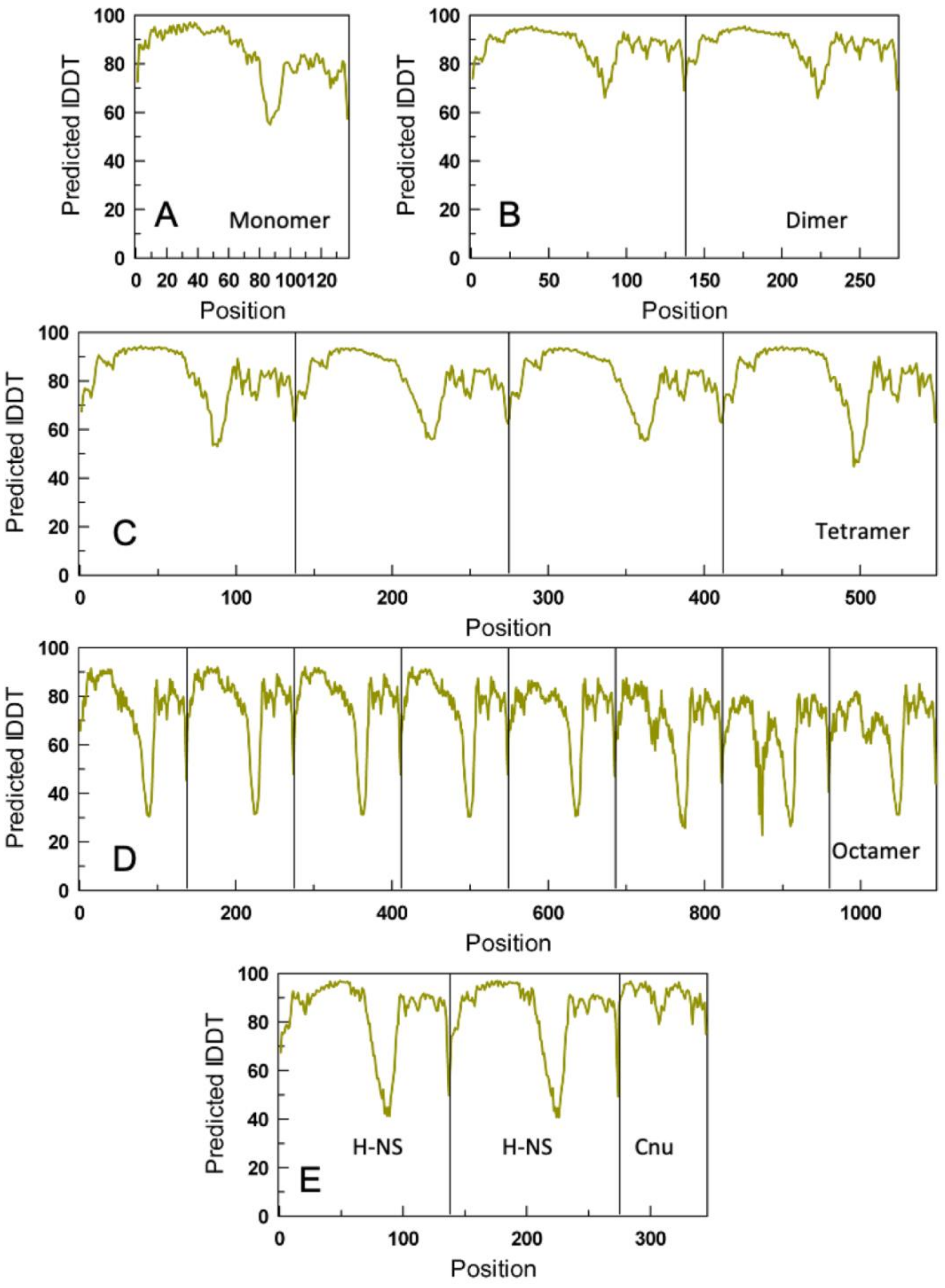

Figure S1 Predicted local distance difference test scores (y-axis) as a function of sequence position for the highest ranked structural model from the AlphaFold-ColabFold interface. Panel A-D show the scores for monomer to octamer models of H-NS. It is clear that the octamer model has a lower prediction accuracy than the monomer, dimer and tetramer models. Panel E shows the model quality for $(H\text{-}NS)_2$:Cnu multimer. Here, it is can be seen that the quality of H-NS dimer is lower than that of the dimer alone.

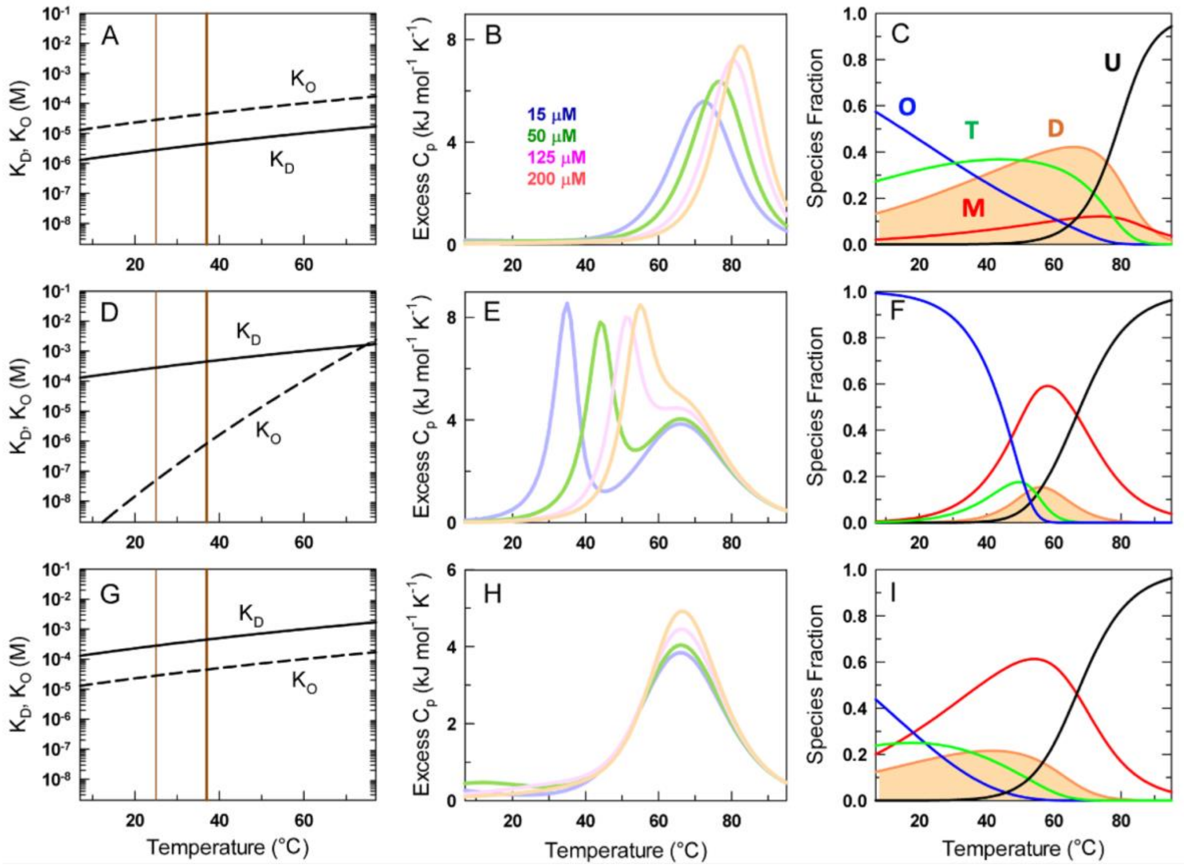

Figure S2 Dependence of heat capacity curves (panels B, E, H) and species fractions (panels C, F, I) on different assumptions of the dimerization (K_D) and oligomerization constants (K_O) (panels A, D, G). The light and dark brown lines in panels A, D, and G highlight 25 ºC and 37 ºC, respectively. The species fractions are displayed for a H-NS concentration of 200 μM with blue for octamer, green for tetramer, orange for dimer, red for folded monomer and black for unfolded monomer (panels C, F, I). (Top Row) Dimerization is stronger than oligomerization but with identical temperature dependence. Here, the experimentally observed double transition (Figure 3A in the main text) is absent in the predicted DSC thermograms. (Middle Row) K_O has a stronger temperature dependence than K_D. Double transitions are observed but since oligomerization is highly favored, the fraction of octameric species approach unity, which is not observed in experiments. Moreover, dimer population is predicted to be zero at 25 ºC while a finite population of dimers observed experimentally under ambient conditions. The first transition reports on the melting of the octamer while the other species dissociate cooperatively in a narrow temperature range. (Bottom Row) Oligomerization is stronger than dimerization but with identical temperature dependence ($K_O < K_D$). The similar magnitudes conspire to result in transitions which are not too sensitive to protein concentrations. The weak dimerization (relative to oligomerization) results in a large population of monomeric species (~40%) at 300 K, which is not observed experimentally.

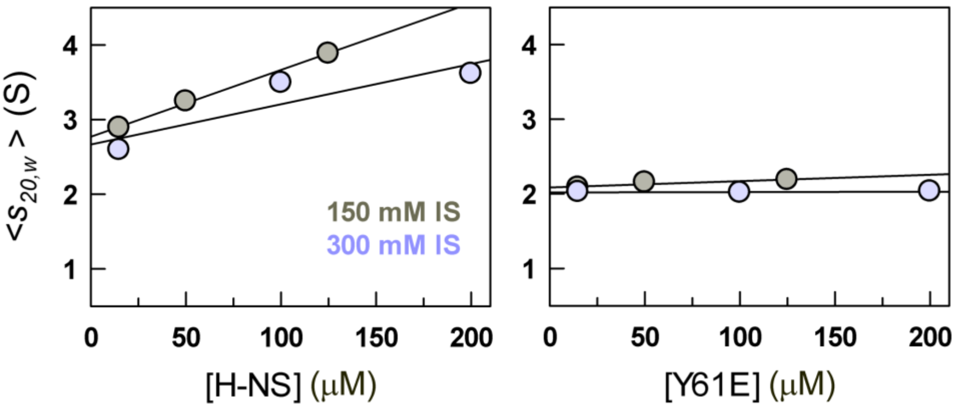

Figure S3 Weight-averaged sedimentation coefficients as a function of ionic strength and protein concentration at pH 7, 20 ºC for the WT H-NS (left panel) and the Y61E mutant (right panel). Note the flat concentration dependence for the Y61E variant.

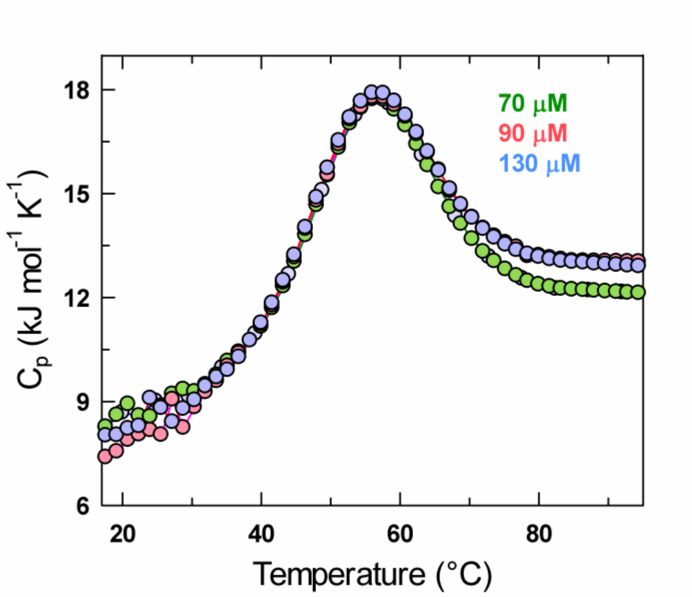

Figure S4 The H-NS DBD exhibits concentration-independent thermal unfolding with a peak heat capacity temperature of 57 ºC.

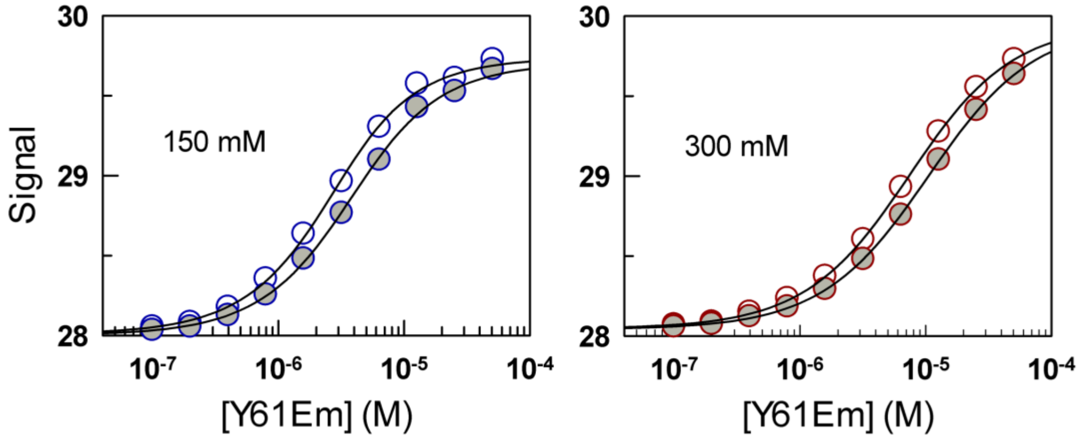

Figure S5 Titration curves of the Y61Em mutant of H-NS (i.e. Y61E/W109F H-NS) on to Cnu at two temperatures (open circles is 25 ºC and filled circles is 37 ºC) and at the two ionic strength conditions indicated. The 'Signal' here represents intensity-averaged wavenumber (see Methods section in the main text).

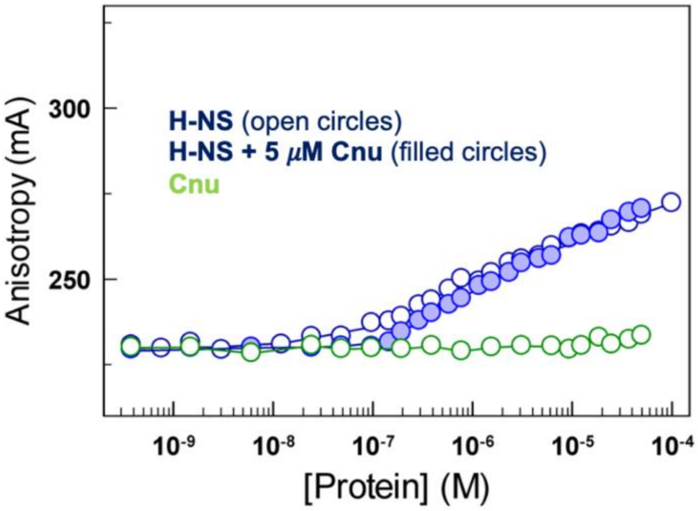

Figure S6 Anisotropy of Cy3-labeled 100-bp DNA as a function of H-NS concentration in the absence of Cnu (blue circles) or H-NS in the presence of 5 μM Cnu (blue filled circles) at 25 ºC, 150 mM ionic strength. Titration of Cnu alone reveals little change in the anisotropy of DNA, indicating that Cnu does not bind DNA.

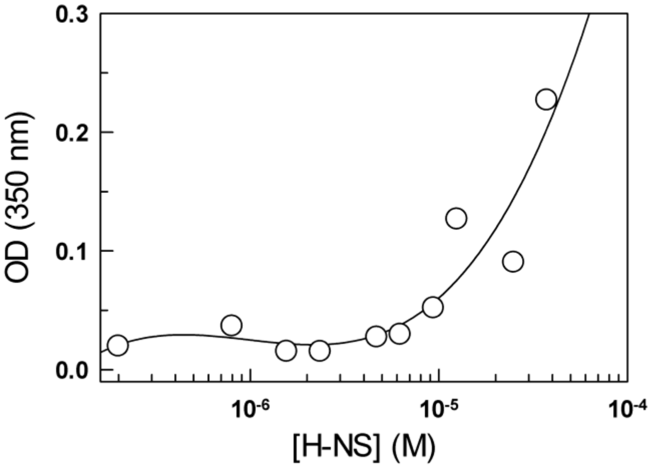

Figure S7 Light scattering at 350 nm as a function of H-NS concentration at a fixed concentration of Cnu (50 μM) and Cy3-labeled DNA (40 nM) at 25 ºC, 150 mM ionic strength. The scattering measurements were averaged over 30 minutes. Standard deviations are smaller than the symbols.

Table S1 Parameters employed for simulating DSC profiles. See Methods section in the main text for a description of the parameters. The shaded columns best capture the experimental observations under the conditions noted. The dissociation constants as a function of temperature can be derived from the parameters below and equation 3 provided in the main text.

	150 mM (Figure 3)	150 mM (Figure S1, Top Row)	150 mM (Figure S1, Middle Row)	150 mM (Figure S1, Bottom Row)	300 mM (Figure 4)
**T_O (K)**	333	333	333	333	333
**ΔH_O (kJ mol^{-1})**	-30	-30	-180	-30	-40
**K_O (M)**	10^{-4}	10^{-4}	10^{-4}	10^{-4}	10^{-4}
**T_D (K)**	333	333	333	333	333
**ΔH_D (kJ mol^{-1})**	-180	-30	-30	-30	-75
**K_D (M)**	10^{-3}	10^{-5}	10^{-3}	10^{-3}	10^{-4}
**T_U (K)**	340	340	340	340	330
**ΔH_U (kJ mol^{-1})**	120	120	120	120	150

Table S2 Predicted dimerization and oligomerization constants from the thermodynamic model.

	150 mM (Figure 3F)		300 mM (Figure 4G)	
**Temperature (^{0}C)**	K_D **(M)**	K_O **(M)**	K_D **(M)**	K_O **(M)**
**13**	2.3826e-8	1.6963e-5	1.1852e-6	9.3903e-6
**25**	5.0069e-7	2.8179e-5	4.2152e-6	1.8474e-5
**37**	8.3127e-6	4.5008e-5	1.3590e-5	3.4492e-5
